# Antimicrobial activity of ion-substituted calcium phosphates: A systematic review

**DOI:** 10.1016/j.heliyon.2023.e16568

**Published:** 2023-05-26

**Authors:** Robert Kamphof, Rui N.O. Lima, Jan W. Schoones, Jacobus J. Arts, Rob G.H.H. Nelissen, Giuseppe Cama, Bart G.C.W. Pijls

**Affiliations:** a Leiden University Medical Center, Department of Orthopaedics, Albinusdreef 2, 2333, ZA, Leiden, the Netherlands; b CAM Bioceramics B.V., Zernikedreef 6, 2333, CL, Leiden, the Netherlands; c Delft University of Technology, Mekelweg 5, 2628, CD, Delft, the Netherlands; d Leiden University Medical Centre, Directorate of Research Policy, Albinusdreef 2, 2333, ZA, Leiden, the Netherlands; e Maastricht University Medical Centre, Department of Orthopaedic SurgeryP., Debyelaan 25, 6229, HX, Maastricht, the Netherlands

**Keywords:** Calcium phosphate, Ion substitution, AMR, DARTBAC, Biomaterial

## Abstract

In this systematic review, the antimicrobial effect of ion-substituted calcium phosphate biomaterials was quantitatively assessed.

The literature was systematically searched up to the 6th of December 2021. Study selection and data extraction was performed in duplo by two independent reviewers with a modified version of the OHAT tool for risk of bias assessment. Any differences were resolved by consensus or by a referee. A mixed effects model was used to investigate the relation between the degree of ionic substitution and bacterial reduction.

Of 1016 identified studies, 108 were included in the analysis. The methodological quality of included studies ranged from 6 to 16 out of 18 (average 11.4). Selenite, copper, zinc, rubidium, gadolinium, silver and samarium had a clear antimicrobial effect, with a log reduction in bacteria count of 0.23, 1.8, 2.1, 3.6, 5.8, 7.4 and 10 per atomic% of substitution, respectively. There was considerable between-study variation, which could partially be explained by differences in material formulation, study quality and microbial strain. Future research should focus on clinically relevant scenarios *in vitro* and the translation to *in vivo* prevention of PJI.

## Highlights

• Evidence on antimicrobial ion-substituted calcium phosphate biomaterials was reviewed using linear mixed effects models.
• Seven ions with clear antimicrobial effect were identified.
• There was considerable between-study variation, partially due to differences in material formulation, study quality and microbial strain.

## Graphical abstract

**Figure:**
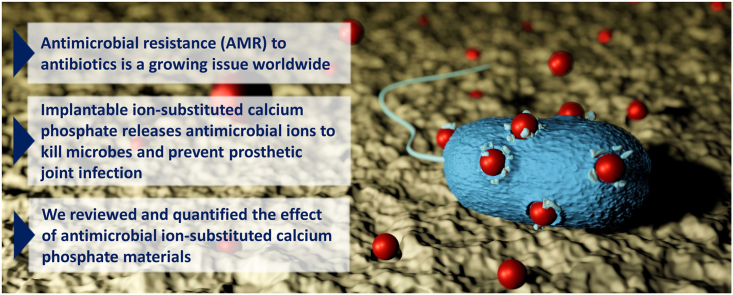

## 1 Introduction

Prosthetic Joint infection (PJI) occurring at the surface of an implant is one of the most devastating adverse event for patients after arthroplasty surgery [1,2]. These infections often cannot be treated by conventional antibiotics due to the formation of bacterial biofilms, which protect the microbes at the implant surface against external threats. Moreover, the efficacy of antibiotics is threatened by growing incidence and severity of antimicrobial resistance (AMR) [3,4]. When treatment fails, implant-related infection will inevitably result in severe patient morbidity and the need for implant removal and extensive debridement surgery to remove the biofilm, followed by months of antibiotic treatment. These procedures are financially costly and can have far-reaching consequences for the patient's quality of life such as extended hospital stay, invasive revision surgery and increased patient mortality [5]. Therefore, there is a need for alternative options to antibiotics for the prevention and treatment of implant-related infection.

Calcium phosphates (CaPs) are a class of ceramic materials that are chemically similar to the mineral component of human bone and dental tissue [6]. Due to this similarity, CaPs are used extensively in the clinic as implant coatings and for bone reparation procedures. Calcium phosphates degrade over time due to dissolution as well as cell-mediated processes, and can be fully replaced by healthy bone [7]. CaPs are applied in a wide array of formulations: as implant coatings, in the form of mouldable putties or injectable CaP bone cement, or as granules or blocks for templated bone regeneration [6]. Porous CaP granules can also be used as a drug delivery device in order to release (antimicrobial) drugs *in situ* [8].

The ions in the lattice of CaP can be substituted for small quantities of other ions [9]. For instance, a variety of ion impurities are present in natural bone, which gives it a high biological activity [6,9]. Strontium and magnesium are examples of ions that are naturally present in bone mineral. As a consequence, several studies have examined the possibility to render CaP antimicrobial by doping these materials with antimicrobial ions such as silver, copper and zinc. CaP implants doped with these ions could help in preventing PJI and biofilm formation, or be used to treat an existing infection [10].

Over the past two decades, the antimicrobial performance of CaPs substituted with a wide variety of ions has been reported. However, few attempts have been made to progress from *in vitro* antimicrobial experiments to an *in vivo* setting. Moreover, no clinical studies have been performed to investigate the potential for these materials to prevent infections in humans. This lack of progress can be partly attributed to the large number of different ions, synthetic procedures and measures of antimicrobial effectiveness. This large variety makes it difficult to compare data and make generalised statement on the antimicrobial effect of ion-substituted CaPs. Therefore, there is a need to summarise the available data on these materials.

A limited number of narrative reviews on the topic of doping antimicrobial ions into CaP have been written in the past, which are subject to a high risk of selection bias [10,11]. No up-to-date reviews exist for this rapidly evolving field of study, and a quantitative approach to review the antimicrobial effect of ion-substituted CaPs is completely absent. Consequently, this review is the first systematic and quantitative review on the antimicrobial potential of ion-substituted calcium phosphates. We aim to answer the question: ‘to what degree can the substitution of ions in the structure of calcium phosphate reduce the growth of bacteria?’ Using the knowledge gained in this study, we hope to facilitate an informed material design process for future studies. In this way, we aim to expedite the clinical application of ion-substituted CaP materials.

## 2 Methods

This systematic review was performed and reported according to the Preferred Reporting Items for Systematic Reviews and Meta-Analyses (PRISMA) guideline [12]. The protocol was registered a priori on the Harvard Dataverse on the 6th of November 2021, at https://doi.org/10.7910/DVN/HEP18U. The final data set was posted on the Harvard Dataverse on the 17th of June 2022, at https://doi.org/10.7910/DVN/Q0OP9D. The search strategy was registered on the Harvard Dataverse on the 4th of July 2022 at https://doi.org/10.7910/DVN/K5LWQV. The studies resulting from the literature search are described in Appendix A. The code used to produce the data for this review was posted on the Harvard Dataverse on the 3rd of August 2022, at https://doi.org/10.7910/DVN/RNKXTP.

### 2.1 Search strategy

The literature search strategy was composed in collaboration with a medical information specialist (JS). The following databases were searched from the date of their inception to the 6th of December 2021: PubMed, Embase (OVID version), Web Of Science, Cochrane Library and Emcare (OVID version). The search strategy consisted of the following components (combination of controlled vocabulary and free text term).

1. The word ‘ion(s)’ or the name of various elements that were identified from a cursory literature search as being used for antimicrobial calcium phosphates,
2. The word ‘substitute(d)’ or ‘dope(d)’,
3. Various terms signifying the use of calcium phosphate materials, such as ‘calcium phosphate’, ‘(hydroxy)apatite(s)’, ‘tricalcium phosphate(s)’ or ‘Brushite(s)’,
4. Various terms implying their use for antimicrobial purposes, such as ‘antimicrobial’, ‘antibacterial’ or ‘anti-infective’.

### 2.2 Literature screening

Two reviewers (RK and RL) independently screened the literature on title and abstract. Both reviewers noted their findings in a pre-designed form. Afterwards, both databases were compared and any disagreements were resolved by consensus or by consulting a referee (BP). The studies remained eligible in case the information in the title and abstract did not suffice or in case of any doubt.

Subsequently, the full text of eligible studies was retrieved and evaluated by two reviewers (RK and RL) independently. Both recorded their notes in a pre-designed form. Any disagreements were resolved by consensus or by consulting a third referee (BP). During both phases all studies were subjected to the in- and exclusion criteria listed below.

Inclusion criteria.

1. Studies reporting *in vitro* or *in vivo* (animal study) antibacterial activity of (co)substituted hydroxyapatite, (α- or β-) tricalcium phosphate or dicalcium phosphate (brushite), as defined in Table 1.

**Table 1:** The phases of calcium phosphate considered in this review.

CaP Phase	formula	Ca: P
Hydroxyapatite (HA)	Ca_10_(PO_4_)_6_(OH)_2_	1.67
α-tricalcium phosphate (α-TCP)	Ca_3_(PO_4_)_2_	1.5
β-tricalcium phosphate (β-TCP)	Ca_3_(PO_4_)_2_	1.5
Dicalcium phosphate (brushite/DCPD)	CaHPO_4_·2H_2_O	1
Dicalcium phosphate anhydrate (DCPA)	CaHPO_4_	1
2. Studies evaluating and quantifying the antimicrobial properties of the material (for example, in terms of colony-forming units).

Exclusion criteria.

1. Studies which do not focus on (co)substituted hydroxyapatite, (α- or β-) tricalcium phosphate or dicalcium phosphate (brushite).
2. Studies that did not provide a quantitative measure of the substituted ion concentration and the antimicrobial activity of these materials.
3. Studies that described composite materials or combinations of calcium phosphate with a different substance (such as a polymer, drug-like molecule or metal nanoparticles).
4. Studies written in a language not spoken by a member of the research team (languages other than English, Dutch, German, French, Spanish, Portuguese and Italian).
5. Studies that did not report the number of experiments upon which the data was based, with the exception of studies that presented no measure of the variance; in that case, the study was included and n was taken to be 1.

### 2.3 Data extraction and data synthesis

Data was extracted in duplo from the included papers independently by two reviewers (RK and RL). The data extraction comprised study characteristics, type and synthesis of Calcium phosphates, characteristics of substituted ions (e.g. charge and concentration), type of micro-organism, method(s) used to determine biofilm formation/bacterial growth/bacterial vitality or reduction thereof and outcomes (e.g. K ratio, percentage reduction or log reduction). Statistical analysis was performed using the R programming language (version 4.2.0) in RStudio (version 2022.02.2 + 485).

To harmonise the extracted data for meta-analysis, some post-processing steps were taken. Weight percentages (wt%) of substituted ions were converted into atomic percentages (at%). The atomic percentage is defined in this case as the proportion of substituted atoms as part of the total number of atoms in the unit cell. Additionally, where possible, results reported as percentage bacterial reduction (the K-ratio), log (CFU) or optical density (OD) were converted to the log reduction in bacteria count, which is the unit in which the meta-analysis was conducted. Log (CFU) reduction was always taken in comparison to unsubstituted CaP, where the data allowed it. The mathematical methods used to convert the units can be found in Appendix B.

The data from the included studies was pooled in a meta-analysis with the random effects model. To obtain the effect sized and corresponding 95% confidence intervals, a linear mixed model was constructed using the lme 4 package for R (version 1.1.29). A linear regression with intercept 0 and variable slope was fitted to the pooled data between the log reduction in CFU count as dependent variable and the atomic percentage of substation as independent variable. Furthermore, the effect of possible confounding factors, culture time and material concentration (defined as the amount of ion-substituted CaP material in the culture medium in mg/mL) by the addition of those variables as secondary independent variables to the model. Since it was assumed that data points from the same study were more closely correlated than between-study data, the study ID was added as a random effect modifier on the slope predicted for the atomic percentage. In summary, the linear models used in this review took the following shape:

Δlog(CFU)=0+at%+(0+at%|ID)

Δlog(CFU)=0+at%+t+(0+at%|ID)

Δlog(CFU)=0+at%+material_conc+(0+at%|ID)

Sources of heterogeneity in the data was explored via subgroup analysis. In particular, the antimicrobial effect of individual ions, the microbe strain, material formulation, and study quality were investigated using subgroup analysis of the data set.

To clarify the effect of individual ions, only the data points corresponding to mono-substituted calcium phosphates were used in the initial model. Because of the large number of possible combinations and ion concentrations of co-substituted materials, the effect of co-substitution was examined on a case-by-case basis. Due to a lack of available data, the effect of the CaP phase could not be included in the statistical model.

### 2.4 Risk of bias assessment

To assess the risk of bias in included studies, an adapted version of the OHAT Risk of Bias Assessment Tool for Human and Animal Studies was used, which was modified for *in vitro* studies (Appendix C). The risk of bias of each included study was scored from 0 (high risk of bias) to 18 (low risk of bias). Funnel plots were generated to assess for possible publication bias in the included studies on zinc- and silver-substituted CaPs. Linear models were fitted to the data of each individual study and grouped for each ion. The resulting slopes and variances were used to construct funnel plots using the metaphor package for R (version 3.4.0). In case of asymmetry in the funnel plots, the trim-and-fill method was used to estimate the magnitude of the bias.

### 2.5 Protocol deviations

The methods employed in this review deviated from the protocol in certain areas. Because no studies were included that contained *in vivo* data, it was not necessary to assess for risk of bias in *vivo* studies. Since almost all studies provided individual data points instead of summary measures (such as means), it was not necessary to construct forest plots or to calculate τ^2^ statistics and I^2^ statistics.

## 3 Results

### 3.1 Study selection

**Fig. 1:**
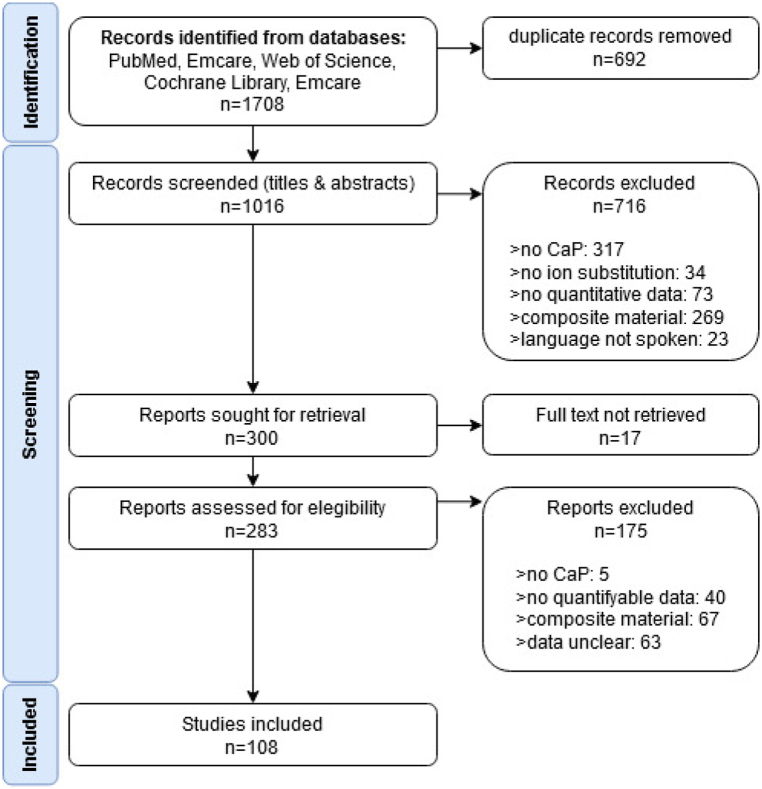
The PRISMA flow diagram for this review.

The literature search was performed on the 6th of December 2021. A total of 1016 journal articles were retrieved. From this initial dataset, 908 were excluded, leaving a final dataset of 108 articles published between 1998 and 2022. The PRISMA flow diagram for this review can be found in Fig. 1. A detailed list of all studies and the reason for their exclusion can be found in Appendix A.

From the included data set of 108 studies, a total of 2183 data points were extracted, each representing a single measurement of the antibacterial effectiveness of an ion-substituted CaP material. None of the included studies reported the antibacterial effect of substituted calcium phosphate materials in an *in vivo* setting.

### 3.2 Study characteristics

A total of 23 different ions were identified that were tested for their antimicrobial effectiveness. The most common ions to be substituted were zinc (n = 31), silver (n = 29) and copper (Cu^2+^/Cu^+^, n = 20). Of the 108 included studies, 93 reported on the effects of mono-substituted CaP, and 33 studies studied co-substituted CaP material.

In terms of material phase, hydroxyapatite is the most frequently studied (n = 95), while other pure or mixed CaP phases were reported 7 or fewer times. The CaPs synthesised in the included articles were tested in the form of (nano)powders or powders pressed into tablets (n = 88), coatings on a metallic substrate (n = 22), as a CaP cement (n = 1) or a porous scaffold (n = 1).

Six different outcome measures of antimicrobial effectiveness were used: the K-ratio or percentage bacterial reduction (n = 37), log colony-forming units (log CFU, n = 46), zone of inhibition/exclusion zone (n = 31), bacterial optical density (OD, n = 6), minimum inhibitory concentration (MIC, n = 6) and minimum bactericidal concentration (MBC, n = 3). Results presented as log CFU, K-ratio or OD were converted to log CFU reduction, giving a total of 78 studies that could be used for meta-analysis. Because of the small number of studies that report the MIC or MBC, these results were not used in further data analysis. Zone of inhibition was reported in an inconsistent manner, so a pooled analysis was not considered to be appropriate. As a result, the data of Zone of inhibition experiments was not used for meta-analysis.

In 93 studies, gram-positive bacteria were tested. Gram-negative bacteria were also tested in 92 studies. The tested bacteria were *E. coli* (n = 84), *S. aureus* (n = 78), *P. aeruginosa* (n = 24). 24 other micro-organisms were tested 10 times or fewer (Appendix A). In 16 articles, the study included the fungal strain C. albicans. 64 of the 108 studies also reported on the toxicity of their materials towards human cells using an assay like the haemolytic ratio, osteoblast proliferation or another methodology.

### 3.3 Antimicrobial effect

#### 3.3.1 The effect of culture time and material concentration

There was a small effect for both the exposure time and powder concentration variables, with an estimated bacterial reduction of 0.011 log CFU per hour of culture time and 0.00029 log CFU per mg/mL of powder added to the culture medium. Since the majority (>80%) of the measurements were performed at or below 24 h culture time, the overall effect of culture time on the predicted outcome is small. Similarly, the powder concentration of the included studies ranges from 0.00025 mg/mL to 250 mg/mL, with a single outlier study at 2500 mg/mL. Because the effect of exposure time and powder concentration on bacterial reduction is relatively small, these variables were excluded from further analysis for the purpose of clarity.

#### 3.3.2 The antimicrobial effect of different ions

**Fig. 2:**
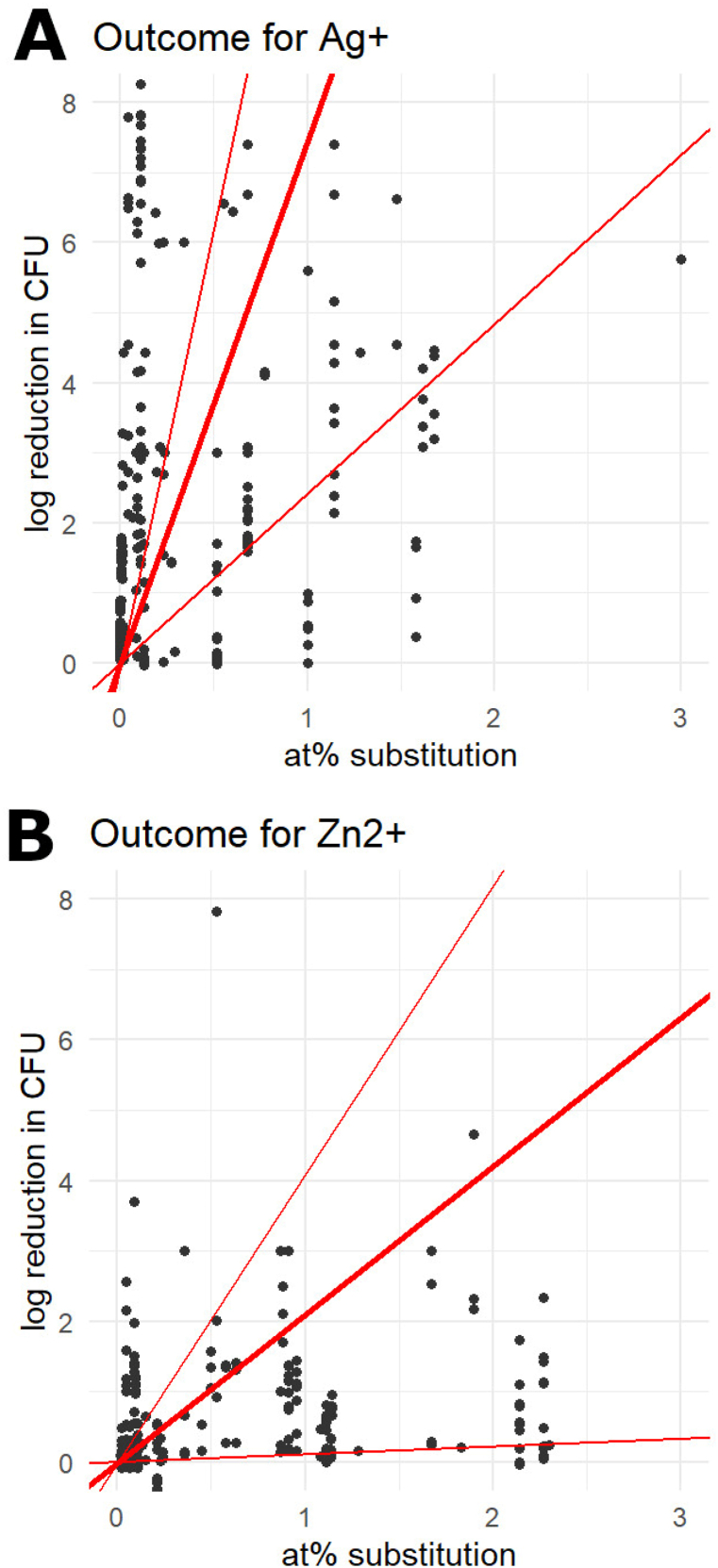
The outcome in log reduction in CFU counts per substituted at% of silver (A) and zinc (B). The thick red line denotes the slope predicted by the mixed effects model, and the thin red lines show the predictions for the lowe- and upper bound of the 95% confidence interval. (For interpretation of the references to colour in this figure legend, the reader is referred to the Web version of this article.)

Ions with clear antimicrobial effects (slopes greater than 0.5, with a 95% CI lower bound above 0) included silver, copper and zinc, and the heavy metals gadolinium, rubidium and samarium.

(Table 2 and Fig. 2A and B). The selenite ion (SeO_3_^2−^)was also considered antimicrobial; as a composite ion of 4 atoms, the reported slope can be considered 4× the value reported in the table.

**Table 2:** The predicted antimicrobial slopes (in log CFU reduction per at% substitution) for each ion. Values in blue were fitted using a simple linear model.

Ion	#studies	Slope	95% CI	
Ag^+^	15	7.44	2.42	12.46
Cu^+^/Cu^2+^	11	1.85	0.26	3.44
Zn^2+^	15	2.11	0.11	4.10
Gd^3+^	1	5.82	3.50	8.14
Rb^+^	1	3.63	3.03	4.23
Sm^3+^	1	10.28	9.72	10.84
SeO_3_^2-^	2	0.23	0.18	0.27
Co^2+^	2	0.18	0.08	0.27
F^−^	3	0.12	0.02	0.22
La^3+^	1	0.47	0.33	0.61
Sr^2+^	5	0.14	0.03	0.26
CO_3_^2-^	2	0.23	−0.21	0.67
Eu^3+^	2	0.26	−0.52	1.04
Fe^2+^/Fe^3+^	1	0.02	−0.01	0.06
Mn^2+^	1	0.03	0.00	0.07
Li^+^	1	0.44	−0.36	1.23
Ti^4+^	1	0.02	−0.10	0.15
SiO_4_^4-^	1	0.04	0.02	0.07
Ce^3+^/Ce^4+^	5	11.49	−10.42	33.40
Mg^2+^	5	1.82	−1.36	5.00

A smaller antimicrobial effect (slopes 0.1–0.5, with a 95% CI lower bound greater than 0) was found for cobalt, fluorine, lanthanum, and strontium. No clear antimicrobial effect was found for carbonate (CO_3_^2−^), europium, iron (Fe^2+^ or Fe^3+^), manganese, titanium, or silicate (SiO_4_^4−^).

**Fig. 3:**
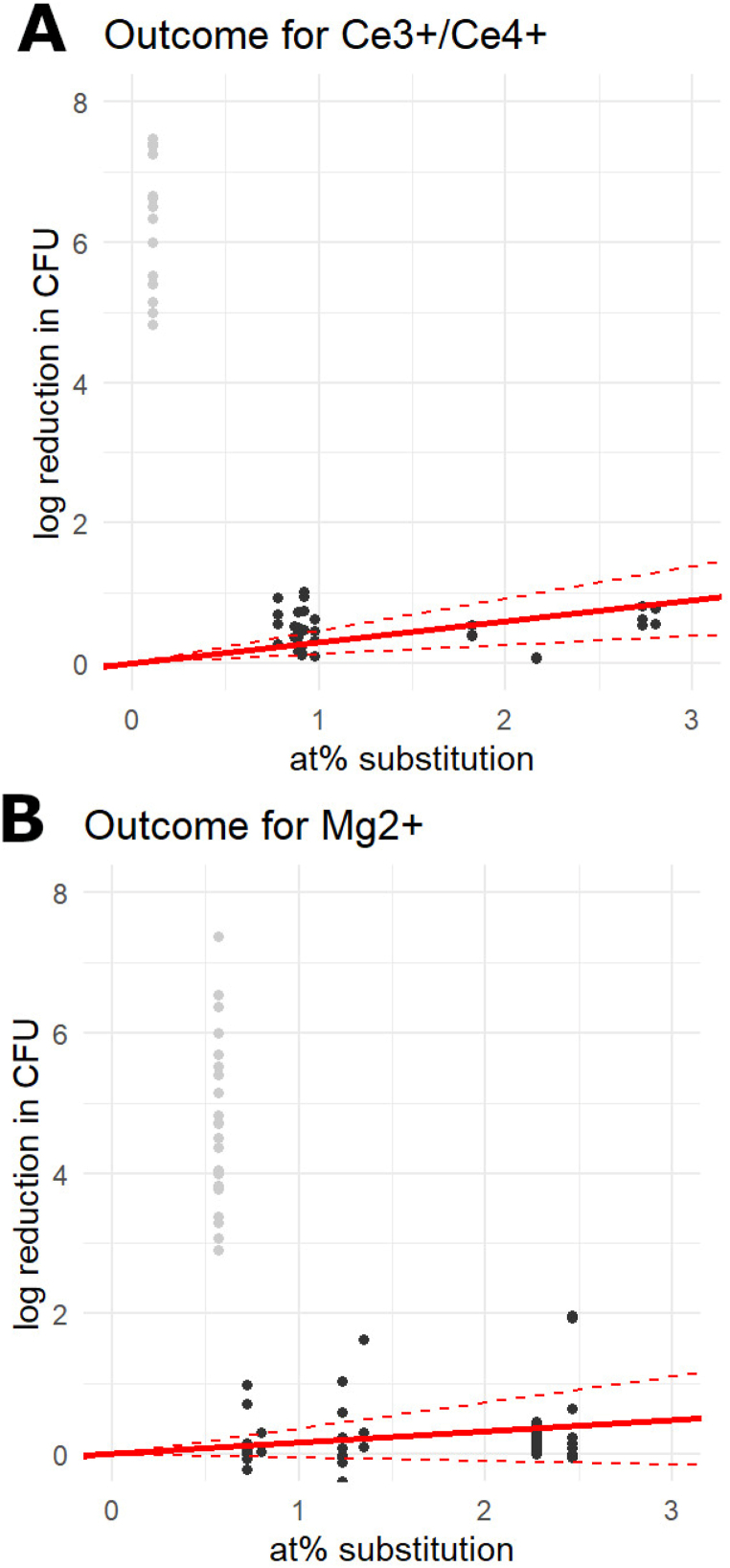
The outcome in log reduction in CFU counts per substituted at% of cerium (A) and magnesium (B). The outlier study in the data of each ion is coloured grey.

Finally, the antimicrobial effect of cerium (Ce^3+^ or Ce^4+^) and magnesium was unclear. The predicted effect size for these ions was high (28 log CFU/at% and 2 log CFU/at%, respectively), but the variance of the data for these ions was so large that the 95% confidence intervals extended well into the negative. A single outlier study was present for both ions, which were published separately by the same primary author (Fig. 3A and B). When these outlier studies were removed, the estimated effect size for cerium was reduced to 0.30 log CFU/at% (95% CI 0.13–0.46), and 0.16 (95% CI -0.05-0.37) for magnesium. Therefore, they can be considered moderately antimicrobial and non-antimicrobial, respectively. Images with the data and the predicted slopes for all ions can be found in Appendix D. No model could be fit for gallium and vanadate (VO_4_^3−^) ions, because the only articles that studied the antimicrobial potential of these ions only reported the zone of inhibition as outcome.

#### 3.3.3 Relation between micro-organism strain and antimicrobial effect

The slopes predicted by the linear mixed effects model showed an increased effect against gram-negative bacteria for silver and selenite, while copper, zinc, gadolinium and samarium all have a greater effect against gram-positive species (Table 3). However, the 95% confidence intervals had significant overlap, indicating that the direction and magnitude of the difference is still uncertain. Several ions lacked data on their antimicrobial activity against the yeast strain C. albicans. From the ions that did have data, silver and zinc had an increased effect against fungal microbes compared to bacteria.

**Table 3:** The predicted antimicrobial slopes (in log CFU reduction per at% substitution) for each antimicrobial ion against gram-negative, gram-positive and yeast cells. Values in blue were fitted using a simple linear model.

ion	Slope (all)	Slope (G-)	Slope (G+)	Slope (Y)	95% CI (G-)		95% CI (G+)		95% CI (Y)		
Ag+	7.44	9.29	6.40	27.55	0.24	18.33	2.83	9.98	18.52	36.58	
Cu+/Cu2+	1.85	1.07	2.76	1.14	0.08	2.06	0.01	5.51	0.57	1.70	
Zn2+	2.11	0.50	1.51	5.91	0.12	0.88	0.30	2.71	−5.21	17.04	
Gd3+	5.82	1.64	10.00	no data	1.31	1.97	6.45	13.54			
Rb+	3.63	3.37	3.89	no data	2.67	4.07	2.89	4.89			
Sm3+	10.28	9.25	12.03	9.55	8.87	9.64	11.29	12.77	9.18	9.92	
SeO32-	0.23	0.31	0.15	no data	0.13	0.50	0.00	0.30			

#### 3.3.4 Relation between material formulation and antimicrobial effect

**Fig. 4:**
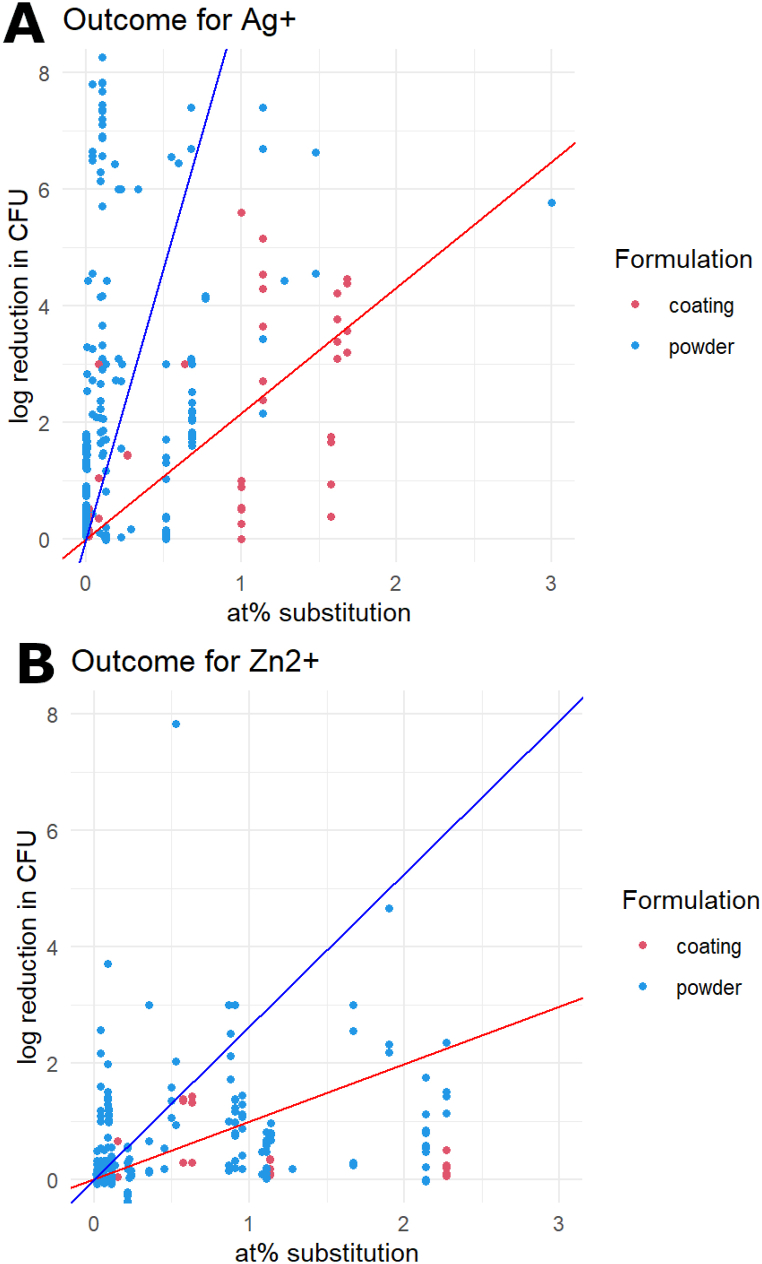
The outcome in log reduction in CFU counts per substituted at% of silver (A) and zinc (B). Testing on CaP coatings generally led to lower outcomes than tests conducted on CaP powders.

Ion-substituted CaPs were most often studied in the form of coatings or (nano)powders in a range of sizes from a few nanometres to several micrometres. As shown in Fig. 4A and B and Table 4, a strong difference was indeed observed between powders and coatings in silver- and zinc-substituted CaP.

**Table 4:** The predicted antimicrobial slopes (in log CFU reduction per at% substitution) for silver and zinc ions substituted into CaP powders and CaP coatings.

Ion	Slope (all)	Slope (powder)	Slope (coating)	95% CI (all)		95% CI (powders)		95% CI (coatings)	
Ag+	7.44	9.33	2.16	2.42	12.46	2.64	16.02	1.05	3.27
Zn2+	2.11	2.63	0.99	0.11	4.11	−0.20	5.47	−0.25	2.24

#### 3.3.5 Effect of co-substitution on antimicrobial effect

**Fig. 5:**
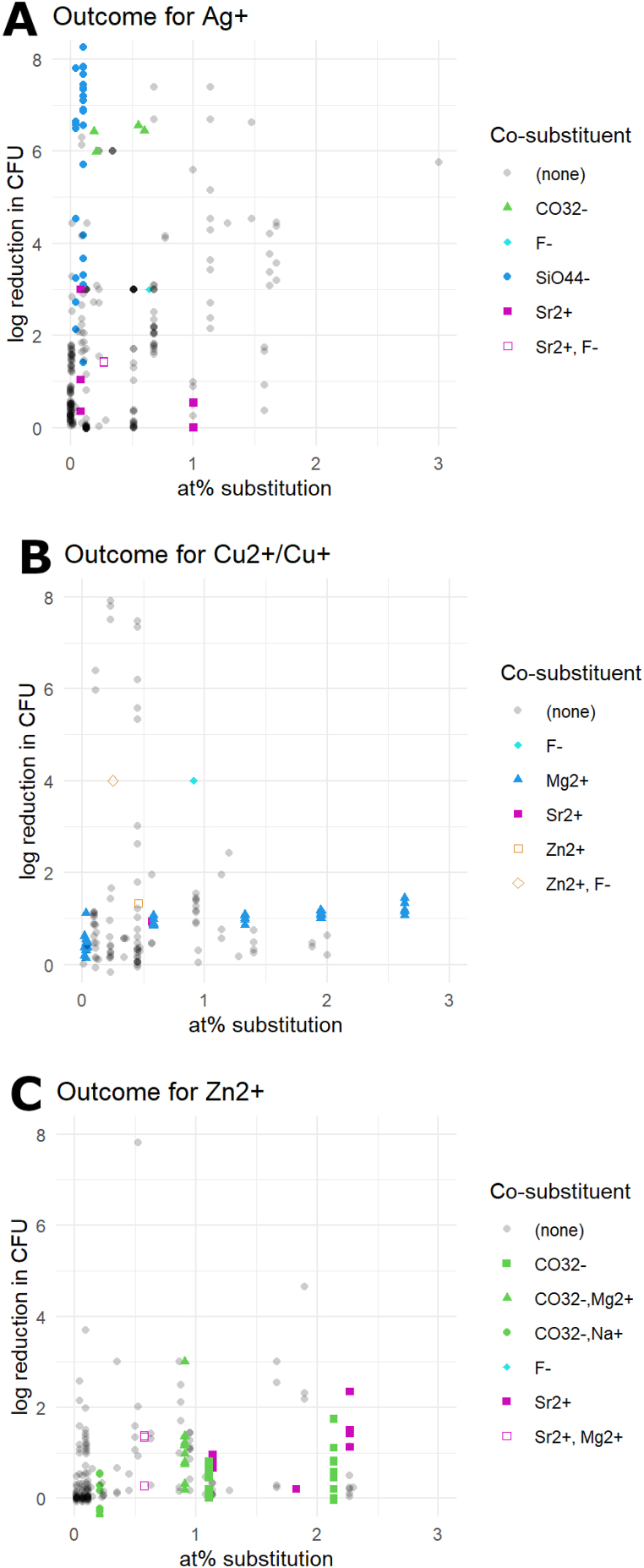
The effect of co-substitution with various ions in CaP substituted with silver (A), copper (B), and zinc (C).

Antimicrobial ions such as zinc, silver and copper, were most frequently combined with non-antimicrobial ions. This was usually done in order to improve their biological properties, such as reducing toxicity of silver, copper and zinc ions, as well as improving the proliferation of human cells (Fig. 5A–C). 33 studies examined co-substituted CaPs. Accordingly, zinc- and copper-co-substituted CaP exhibited no difference in antimicrobial effect compared to mono-substituted materials. However, for silver there was a sharp increase in antimicrobial effect when combined with silicate or carbonate ions, despite the lack of antimicrobial effect of these particles. There was no change in antimicrobial effect when silver was combined with strontium or fluorine.

### 3.4 Risk of bias assessment

The RoB scores given to studies ranged from 6 to 16, with an average of 11.4. The full list of all studies and the resultant RoB scores can be found in Appendix E. An analysis of the (potential) sources of bias can be found in Appendix F.

### 3.5 The effect of bias on the reported outcome

Sensitivity analyses on the possible effect of risk of bias on the results was restricted to silver and zinc, because these ions had sufficient number of studies to allow for such analyses. There were insufficient number of studies for the remaining ions.

The antimicrobial effect was greatest in studies with a lower risk of bias (RoB score 12 or higher). In these studies, the antimicrobial effect was more than two times as large for silver and almost seven times as large in the case of zinc, when compared to studies with a high risk of bias (RoB score 11 or lower). An in-depth explanation of the methods and results that were used for the estimation of bias can be found in Appendix F.

The constructed funnel plots (Appendix F) were asymmetric, suggesting possible publication bias. Hence, trim-and-fill analyses were required. These analyses suggest that antimicrobial effects were overestimated by approximately 15% on average.

## 4 Discussion

This review presents a comprehensive overview of the available data on antimicrobial ion-substituted CaPs. Our analysis of data from 108 studies identified seven ions with antimicrobial effect: silver, copper, zinc, gadolinium, rubidium, samarium and selenite. Furthermore, we observed several characteristics of the CaPs and micro-organisms as well as variables on the study level that modified the observed antimicrobial effect for better or for worse.

No overall effect was found for exposure time and powder concentration on bacterial growth. In contrast to this, within studies that measure the antimicrobial effect at different times or powder concentrations, an effect for these variables is usually observed. This discrepancy is most likely due to large between-study variation in outcome combined with relatively small spread in time and powder concentration between studies, obfuscating the effect of culture time and material concentration in the meta-analysis. The median reported powder concentration is 5 mg/mL, and 82% of all data points were measured at 24 h of culture time or less. Future research must investigate the influence of these parameters further over a wider range of values.

PJI is mainly caused by gram-positive cocci such as *S. aureus* or S. epidermidis [13]. Our models show an increased antimicrobial effect for copper, zinc, gadolinium and samarium against gram-positive microbes, while silver and selenite were found to be more effective against gram-negative species. These differences should be considered during the material design phase. Generally, ions with greater effect against gram-positive strains are better suited to prevent PJI in most cases. However, an ion-substituted CaP material that is part of infection treatment (for example, when an implant has to be replaced with a new one due to infection) can be tailored to contain ions that are particularly suited to combat the microbe that is causing the infection.

Since the surface area of coatings is far smaller than that of powders, it can be expected that ion-substituted coatings have a smaller antimicrobial effect than a powder under the same experimental conditions, since a smaller surface area releases ions at a slower rate. The results of this review support that hypothesis, with a clear increased effect for CaP powders compared to coatings. There are 22 included studies that investigate the antimicrobial effect of ion-substituted CaP coatings, versus 88 studies that examine CaP powders. This is despite the fact that coatings and other ‘macro-materials’ like scaffolds are usually the intended application for ion-substituted CaP. This apparent disconnect with the application presents a weakness of the current evidence base and a source of uncertainty in our models.

No increased antimicrobial effect was found for co-substituted CaP compared to mono-substituted materials, with the exception of silver-substituted CaP that was co-doped with silicate or carbonate. Before considering possible explanations for this improved effect, it is important to note that all 4 studies on silver/silicate co-substituted CaP were published by the same primary author. Therefore, the possibility remains that the observed increased effect for silver/silicate co-substituted CaP is simply caused by methodological differences, rather than the silicate ion itself. However, it is also possible that co-substitution with silicate or carbonate ions provide a synergistic effect. For example, co-substitution with these ions could lead do a decreased material crystallinity and an increased solubility, increasing the release of silver ions from the material.

The study quality was found to be strongly correlated to a large antimicrobial effect. It is currently unclear which aspect of the study quality influences the outcome. Potentially, the (lack of) material characterisation can explain a part of the difference. Studies received a lower RoB score when material characterisation, such as elemental analysis, was lacking or insufficient. For studies without an exact elemental composition, the degree of substitution was calculated based on the ratio of starting reagents. From studies that provide both the ‘predicted’ and ‘measured’ composition, we know that the former is often an over-estimation the real ion concentration, leading to a lower-than-average antimicrobial effect. All future publications should include elemental analysis in order to prevent this kind of uncertainty in the material composition.

### 4.1 Defining the therapeutic window for antimicrobial ions

The therapeutic window of each ion is determined by the antimicrobial effect and by the material's toxicity. Therefore, it is important to assess the substitution limit for different ions to find the maximum antimicrobial effect that it can achieve within the therapeutic window when they are used *in vivo*. Because of the variety of methodologies that are used to measure the biocompatibility of ion-substituted CaP, the results of toxicity experiments conducted by included studies are examined in a qualitative manner.

It should be noted that the majority of included articles studied the properties of ion-substituted CaP powders, so the toxicity of CaPs is also mostly derived from CaP in powder form. Since our results suggested a difference in antimicrobial effect between CaP coatings and powders, it is to be expected that the toxicity of the materials will also differ between different formulations. The exact relation between the material formulation and its antimicrobial and toxic properties should be investigated in future research.

#### 4.1.1 Silver

Silver is one of the most frequently studied ions in substituted CaPs, with well-known strong antimicrobial properties. However silver is also known for its cytotoxic properties against human cells and tissues [14]. 14 included articles reported on the toxicity of silver-substituted CaP with a concentration range of 0.000001–1.7 at%. Among mono-substituted materials, nearly every article showed an increased cytotoxicity (expressed in a reduction of cell viability or increase of the haemolytic ratio) of silver-substituted CaP compared to pure hydroxyapatite [[15], [16], [17], [18], [19], [20], [21], [22]]. Articles that measured the cytotoxicity at multiple time intervals generally reported that the cytotoxicity for silver-substituted CaP is lower than that of pure CaP at short time intervals, but that the toxicity then increased as more time passed. A single paper reported a higher haemolytic ratio for pure HA than for silver-substituted HA with an atomic percentage between 0.05% and 0.1% [18]. According to the authors of the article, the higher haemolytic ratio of pure HA in their findings was caused by its high crystallinity in comparison to silver-substituted HA. This trend between crystallinity and haemolytic effect has been shown before in literature [23]. As such, it might be possible to reduce the cytotoxicity of silver ions by reducing the crystallinity of the material.

In 6 studies, silver was co-substituted with strontium or silicate ions in order to offset the cytotoxicity of silver in the concentration range between 0.04 at%-1at% [[24], [25], [26], [27], [28], [29]]. It was shown that co-substitution with these ions was able to reduce the cytotoxicity of silver in each study. In the range of 0.04 at% to 0.27 at% substituted silver ions, it was possible to fully compensate the cytotoxic effect of silver using co-substitution [25,27].

A limited number of studies are available that report on the *in vivo* safety of silver-doped CaPs. Firstly, one group published three small-scale studies on the *in vivo* performance of silver-doped CaP coatings (∼5 wt% silver, ∼1 at%) in a rabbit model [[30], [31], [32]]. These studies reported no toxicity due to silver while the number of infections was reduced. Secondly, a case study of 50 patients implanted with implants coated with silver oxide (∼0.3 wt% silver, ∼0.06 at%) doped hydroxyapatite was also published [33]. Because the silver in these coatings is present in the form of silver oxide, it falls outside the scope of this review. Still, it is worth noting that no toxic effects due to silver were found for the coated implants.

#### 4.1.2 Copper

Copper exhibits toxicity against humans despite being an essential trace element [34,35]. 7 articles reported on the (non-)cytotoxicity of copper-substituted CaP in a quantitative manner in a copper concentration range of 0.02 at% to 2.6 at%. Of the 5 studies that discuss mono-substituted CaP (0.1 at% to 2 at% of copper), 4 reported an overall negative effect on cell growth [[36], [37], [38], [39]]. However, a single study reported a positive effect of copper substitution on the growth of MC3T3-E1 pre-osteoblast cells at a copper concentration of 0.4 at% [40].

Co-substituted CaP incorporating a mixture of copper and magnesium ions was tested for toxicity in two different studies (0.02 at% to 2.6 at% of copper) [39,41]. Both of these studies found that magnesium was capable of compensating the negative effect of copper to some degree. A copper concentration of 0.02 at% to 0.6 at% could be compensated by equal amounts of magnesium ions; higher copper concentrations were still cytotoxic despite increasing amounts of magnesium ions. Finally, a single study found that a co-substituted CaP material that incorporated both copper (0.5 at%) and zinc (0.4 at%) had an overall net positive effect on the growth of MCT3T3-E1 pre-osteoblast cells [42].

#### 4.1.3 Zinc

As an essential trace element, zinc is not usually associated with toxicity. Nevertheless, studies have shown that high concentrations of zinc can be toxic to human cells *in vitro* and *in vivo* [43]. From the included articles, 17 studies reported on the cytotoxicity of zinc-substituted CaP over a concentration range of 0.022 at% to 3.3 at% zinc. A majority of these articles across the entire concentration range found an overall positive effect on cell proliferation for zinc-substituted CaPs compared to pure CaP for both mono-substituted materials and materials combined with magnesium, carbonate, strontium or copper ions [42,[44], [45], [46], [47], [48], [49], [50], [51], [52]]. Likewise, a different study found no haemolytic effect for zinc-substituted CaP at concentrations ranging from 0.4 at% to 1.7 at% [53]. Two studies reported that the positive effect reverses at higher zinc concentrations, although the cut-off point differed wildly (1.1 at% vs 0.2 at%) [54,55]. In contrast to the other studies, two articles found an overall negative effect of Zinc-substituted material compared to pure CaP at low percentages of zinc substitution (0.05 at% to 0.15 at%) [36,56]. Furthermore, a single study investigated the toxicity of CaP with a very high concentration of Zinc substitution (3.3 at%) at different concentrations of the CaP powder and found that the effect on MC3T3-E1 human pre-osteoblast cells differed strongly depending on the concentration of powder in the culture medium [57]. According to this study, higher powder concentrations were strongly cytotoxic while low concentrations promoted the proliferation of the cells. This suggests that the overall effect of zinc on human cells can be positive or negative based on the exact amount of zinc ions in the culture medium. Future research on zinc-substituted CaPs should focus on identifying this window of opportunity and exploit it to create biomaterials that are both antimicrobial and promote the proliferation of human cells.

#### 4.1.4 Gadolinium

A single included study discussed the antimicrobial potential of gadolinium-substituted HA at three different concentrations between 0.10 at% and 0.25 at% [58]. The same study found that doping gadolinium ions improved the osteoblast proliferation compared to pure HA at all concentrations. These results are surprising, as gadolinium is generally considered to be a toxic heavy metal [[59], [60], [61]]. Further research should focus on verifying these findings and expanding our knowledge on the antimicrobial potential of gadolinium-substituted CaP.

#### 4.1.5 Samarium

The single article on the antimicrobial effectiveness of samarium-substituted CaP did not report a test of the cytotoxic effect of the synthesised material. Because of this, there is no direct evidence of the (non-)toxicity of samarium-substituted CaP. The toxicity of samarium in more general studies was reported to be similar to other lanthanide ions [60,61]. Considering the lack of toxicity that was found for gadolinium-substituted CaP, it is possible that samarium ions can be safely substituted into CaP biomaterials. As the antimicrobial activity for samarium seems very promising, based on the single study that has been conducted so far, further research should focus on expanding the evidence-base on the antimicrobial performance of samarium-substituted CaP as well as its toxicity towards mammalian cells.

#### 4.1.6 Selenite

Many beneficial properties are ascribed to selenium and its ions, selenite (SeO_3_^2−^) and selenate (SeO_4_^2−^). Selenium-based biomaterials can kill bacteria and cancer cells while promoting osteoinduction [62]. However, selenium can become toxic at high concentrations despite being an essential trace element [63].

Among the included studies, 3 examined the bioactivity of selenite-substituted CaP towards human cells compared to unsubstituted HA in the concentration range of 0.07 at% to 9.3 at% [62,64,65]. All studies reported a negative effect of the doped material compared to pure HA at all selenite concentrations, in the form of an increased haemolytic ratio or reduced viability of human fibroblast cells and MG-63 osteoblast-like cells. The latter result is not surprising, because despite often being used as a model for human osteoblasts, the MG-63 cell line is derived from osteosarcoma cells, and should thus be killed by the anti-cancer properties of selenite. Thus, it seems a poor model for the viability of human cells in this case. Regardless, it seems that selenite is toxic to human cells at all the tested concentrations.

A single article examined the viability of MG-63 cells on selenite (1.1 at%) and strontium (0.9 at%) co-substituted into HA [64]. Strontium was able to offset the toxic effects of selenite at lower concentrations (up to 200 μg/mL) of the ion-substituted powder. However, toxicity was still reported at a higher powder concentration. A possibility for future research is to test the toxicity of selenium-substituted CaP that is co-substituted with large amount of strontium, to see if the toxic effect can be balanced at greater concentrations of the CaP powder.

#### 4.1.7 Rubidium

Two studies included data on the biocompatibility of rubidium-substituted CaP. One study examined the effect of rubidium substitution into CaP on the growth of MG-63 human osteosarcoma cells at concentrations between 0.02 at% and 0.2 at% [66]. Increasing the concentration of rubidium ions up to 0.07 at% increased the viability of the human cells, after which the positive effect decreased again. At concentrations higher than 0.15%, the ion-substituted material exhibited a cytotoxic effect on the human cells. The second article reported a small net positive effect on the viability of human HFB4 osteoblasts as the rubidium concentration increased from 0 at% to 1.8 at% in co-substituted CaP incorporating 9 at% of selenite ions [67]. However, the presence of a high concentration of selenite combined with the lack of an appropriate pure CaP control make it difficult to draw a conclusion from these results.

### 4.2 Limitations & strengths

We should consider some of the limitations of the models presented in this meta-analysis. First, our prediction of the effect of gadolinium, rubidium and samarium - all of which are considered to be strongly antimicrobial - is based on a single study for each ion. To confirm the antimicrobial effect of these ions, more research should be conducted on CaP substituted with these ions. Because results reported as zone of inhibition, MIC or MBC were ultimately not used for meta-analysis, there is a large amount of data (∼41% of all data points) that was not used to generate results. Furthermore, there is considerable heterogeneity in the available data; in terms of the reported data and in the variety of synthetic methods and outcome assays. This heterogeneity complicates predictions of the antimicrobial properties of a given CaP material. The resulting uncertainty is exemplified by the wide confidence intervals along the regression lines of the model. Thus, our models can only give a rough estimation of the expected effect size of ions on bacterial growth. An additional limitation is the time between the initial literature search and the publication of the results. However, despite this relatively long timeframe, we do not expect more recent experimental data to change the main outcomes of this review. Finally, since only *in vitro* studies were included in the review, it is uncertain whether our results translate into effective infection prevention *in vivo*.

Despite these limitations, this work presents the first systematic review and meta-analysis that quantitatively pools data from this field of research. Previous narrative reviews on antimicrobial CaPs were only able to qualitatively assess the ions with potential antimicrobial properties, but such reviews are unable to provide quantitative analysis and are subject to selection bias. In this work, we used a systematic approach to summarise the knowledge on antimicrobial ion-substituted CaPs. In line with the AMSTAR criteria for systematic reviews, this review was registered a priori and uses pre-specified reproducible search terms and in/exclusion criteria [68]. The selection process and data extraction were carried out by two independent researchers with a referee to minimise the effect of mistakes and/or selection bias. The presence and impact of bias in the dataset was assessed as well. Furthermore, our analysis is based on validated statistical models, and our source data and code is freely available online.

### 4.3 Future outlook

CaPs are already extensively used in the clinic as bone remodelling materials and osteoinductive implant coatings. Moreover, porous CaP granules loaded with antimicrobial agents are used to fight infection already. However, the potential of ion-substituted antimicrobial CaPs that do not use antibiotics remains untapped so far despite the large number of *in* vitro studies. Few *in vivo* studies exist and no clinical studies have been performed. To regain lost momentum, it is imperative that the field moves towards a more informed material design strategy, in terms of the ions used and the ion concentration, as well as using more standardised antimicrobial tests. This review can help scientists in the study design process. From there on, future research should be focused progressing towards *in vivo* studies.

## 5 Conclusion

The results of our systematic review have shown that silver, copper, zinc, gadolinium, rubidium, samarium and selenite ions have strong antimicrobial effects. The percentage of ion substitution within the biomaterial was the most important determinant of the antimicrobial effect. Other relevant factors to the antimicrobial effect were the material formulation, the used microbial strain and the study quality. Future *in vitro* studies should focus on clinically relevant scenarios and *in vivo* studies are needed to determine whether these *in vitro* effects translate into prevention of PJI *in vivo*.

## Author contribution statement

All authors listed have significantly contributed to the development and the writing of this article.

## Funding & competing interest

This publication is part of the project DARTBAC (with project number NWA.1292.19.354) of the research programme NWA-ORC which is (partly) financed by the Dutch Research Council (NWO).

This article was written in collaboration with CAM Bioceramics B·V., situated in Leiden, The Netherlands. CAM Bioceramics B.V. is a contract developer and manufacturing company of Orthobiologic Calcium Phosphates.

## Data availability

The data that support the findings of this study (search strategy, research data set, code) is openly available in the Harvard Dataverse at https://doi.org/10.7910/DVN/K5LWQV, https://doi.org/10.7910/DVN/Q0OP9D and https://doi.org/10.7910/DVN/RNKXTP.

## Appendix A - reviewed literature

Appendix B - method for data postprocessing Appendix B - method for data postprocessing

Appendix C - method for RoB assessment Appendix C - method for RoB assessment

Appendix D - linear models Appendix D - linear models

Appendix E - RoB assessment Appendix E - RoB assessment

Appendix F - the effect of bias and study quality Appendix F - the effect of bias and study quality
