# Supplementary material for: Antimicrobial activity of ion-substituted calcium phosphates: A systematic review
Source: Heliyon. 2023 May 26;9(6):e16568. doi: 10.1016/j.heliyon.2023.e16568 (PMC10248076; doi:10.1016/j.heliyon.2023.e16568)
Supplement: Appendix C - method for RoB assessment [file mmc2.docx]

# Appendix B: post-processing of data

To homogenise the data resulting from the data extraction, some post-processing steps were taken. The concentration of substituted ions was preferentially reported in atomic percentage, defined as

$${at\%}_{x}=\frac{N_{x}}{N_{total}}*100\%$$

where N_x_ is the number of substituent atoms in the CaP lattice and N_total_ is the number of atoms in the entire lattice. The at% was taken from elemental analysis data where possible. When this data was not provided in the article, the at% was calculated from the intended substitution rate outlined in the methods section. When no information was provided otherwise, it was assumed that the substituent ion replaced calcium (for cations), phosphate (for most anions) or hydroxide (for fluorine ions) in a 1:1 ratio, even if that would not respect charge neutrality. This assumption is in line with many of the included studies.

In papers where the concentration of substituted ions were reported in weight percentage, the atomic percentage was calculated from that number. For this, the following definition of the weight percentage was used:

$${wt\%}_{X}=\frac{{mass}_{x}}{{mass}_{total}}*100\%=\frac{N_{x} / {Mw}_{x}}{{Mw}_{CaP}-{N_{x}*Mw}_{SI}+N_{x}*{Mw}_{x}}*100\%$$

Where N_x_ is the number of substituent atoms in the CaP lattice and Mw_x_, Mw_CaP_ and Mw_SI_ are the molar weights of the substituent ion, the unsubstituted CaP lattice and the ion that is replaced in the lattice, respectively. For multi-substituted materials with more than one substituent, the same formula was used. Furthermore, when polyatomic ions, such as CO_3_^2-^ or SiO_4_^4-^, were reported as the weight percentage of one of its constituent atoms (e.g., wt% C or Si), the reported weight percentage was first converted to the proper number for the entire ion.

For studies where the outcome was measured in optical density experiments, the results were translated into the percentage bacterial reduction (K-ratio) where possible. This was done according to a formula that is already used in several included studies:

$$K=\frac{{OD}_{ref}-{OD}_{sample}}{{OD}_{ref}}*100\%$$

Where OD_sample_ is the optical density of the measured substituted calcium phosphate, and OD_ref_ is the optical density of unsubstituted CaP if reported in the paper. For studies where the OD of unsubstituted materials was not reported, the OD value reported for the control experiment was used for OD_ref_ instead.

For studies where the outcome was measured in colony forming units (CFU), the results were also translated into the K-ratio. Since the difference in log CFU values corresponds to a reduction, K can be calculated according to the following formula:

$$K=\frac{{10}^{log(CFU_{ref})}-{10}^{log(CFU_{sample})}}{{10}^{log(CFU_{ref})}}*100\%$$

Where CFU_sample_ is the number of colony-forming units of the substituted calcium phosphate and CFU_ref_ is the number of colony-forming units of unsubstituted CaP if available, and the number of colony-forming units of the control experiment if not.

The K-ratio can be converted to the Δlog(CFU) using the following formula:

$$\Delta\log\left( CFU \right)=-log(1-\frac{K}{100\%})$$

When a K-ratio of 100% was reported, the Δlog(CFU) was directly taken from the log(CFU) data (when available), or set to Δlog(CFU)=3 (in the case of data originally reported as OD or K-ratio).
