# Supplementary material for: Antimicrobial activity of ion-substituted calcium phosphates: A systematic review
Source: Heliyon. 2023 May 26;9(6):e16568. doi: 10.1016/j.heliyon.2023.e16568 (PMC10248076; doi:10.1016/j.heliyon.2023.e16568)
Supplement: Appendix D - linear models [file mmc3.docx]

# Appendix C: method for risk of bias assessment

To our knowledge, no widely accepted tool exists for assessing the risk of bias in *in vitro* studies. To determine the measure of risk for bias in the *in vitro* articles included in the final dataset, the OHAT Risk of Bias Rating Tool for Human and Animal Studies was adapted to be applicable to *in vitro* work. More specifically, questions 1, 2, 5, 6 and 7 of the OHAT RoB tool were removed, because they are not relevant for *in vitro* studies. The remaining questions, 3, 4, 8, 9, 10, and 11 were assigned a score from 0 (risk definitely high) to 3 (risk definitely low). The total score from 0 to 18 was a measure of the risk of bias in a study. The resulting scores for each included study can be found in Appendix E.

Question 3, d*id selection of study participants result in appropriate comparison groups?*, was used to determine whether the quantitative data reported by the included studies was compared to an appropriate control group, namely unsubstituted calcium phosphate material of the same phase as the substituted samples.

Question 4, *did the study design or analysis account for important confounding and modifying variables?*, was used to check whether sufficient analysis of the measured material and the control material was performed, such as X-ray diffraction, SEM microscopy and ftIR analysis.

The score for question 8, *can we be confident in exposure characterisation?*, was determined by whether the study had measured the ion concentration in the synthesised material, as opposed to simply mixing a certain ratio of reagents, as well as reported the concentration of the synthesised material in the culture medium.

Question 9, *can we be confident in the outcome assessment?*, was used to rate the validity of the method and whether the outcome measure was free of potential bias.

Question 10, *were all measured outcomes reported?*, was used to check if studies had published a study protocol before publication, and whether a study had missing values in the results section.

Finally, the score for question 11, *were there no other potential threats to internal validity*, was determined by the statistical validity of the study, e.g. the sample size of the measured outcome as well as the presence or absence of variance calculations such as the standard deviation or confidence intervals.
