# Supplementary material for: Antimicrobial activity of ion-substituted calcium phosphates: A systematic review
Source: Heliyon. 2023 May 26;9(6):e16568. doi: 10.1016/j.heliyon.2023.e16568 (PMC10248076; doi:10.1016/j.heliyon.2023.e16568)

## Ions with clear antimicrobial effect


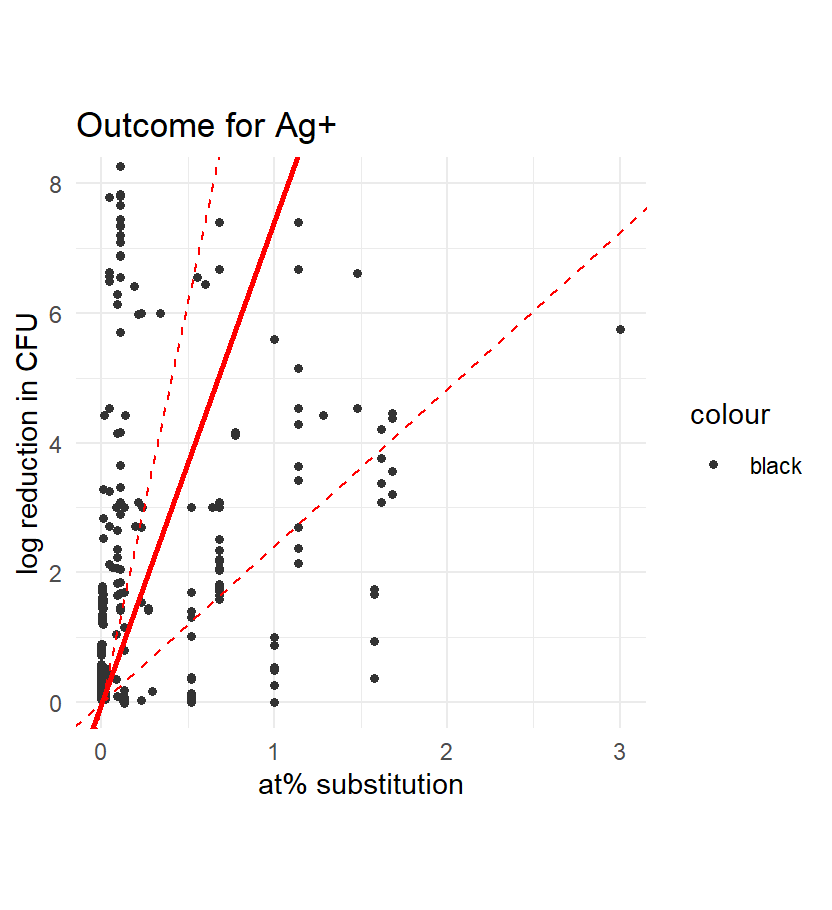

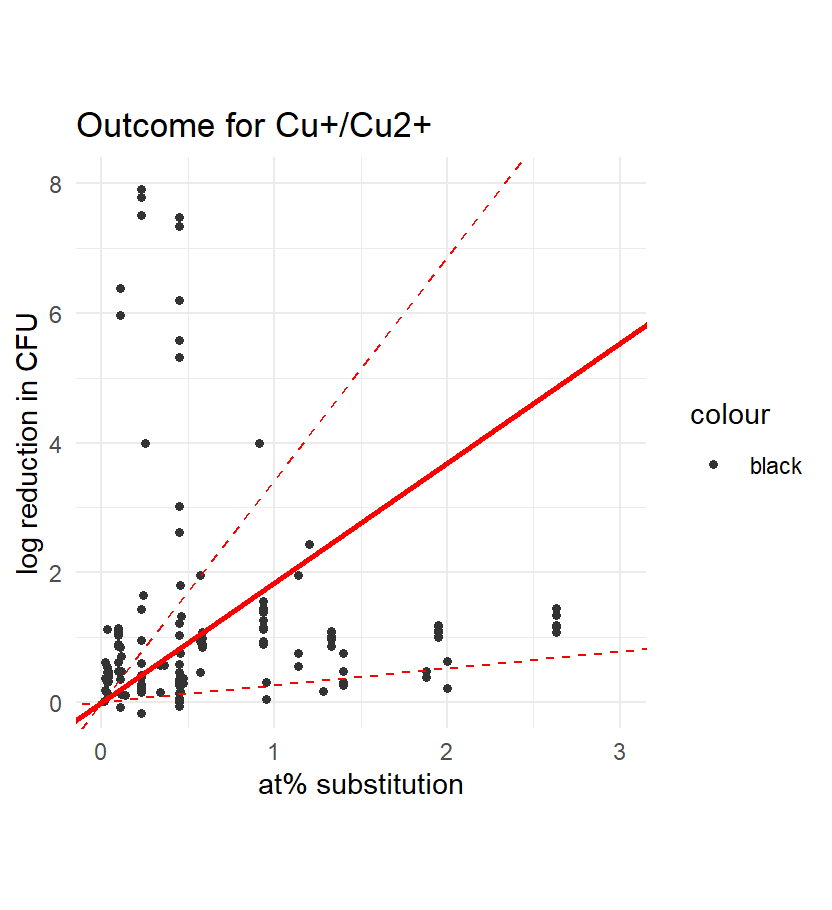


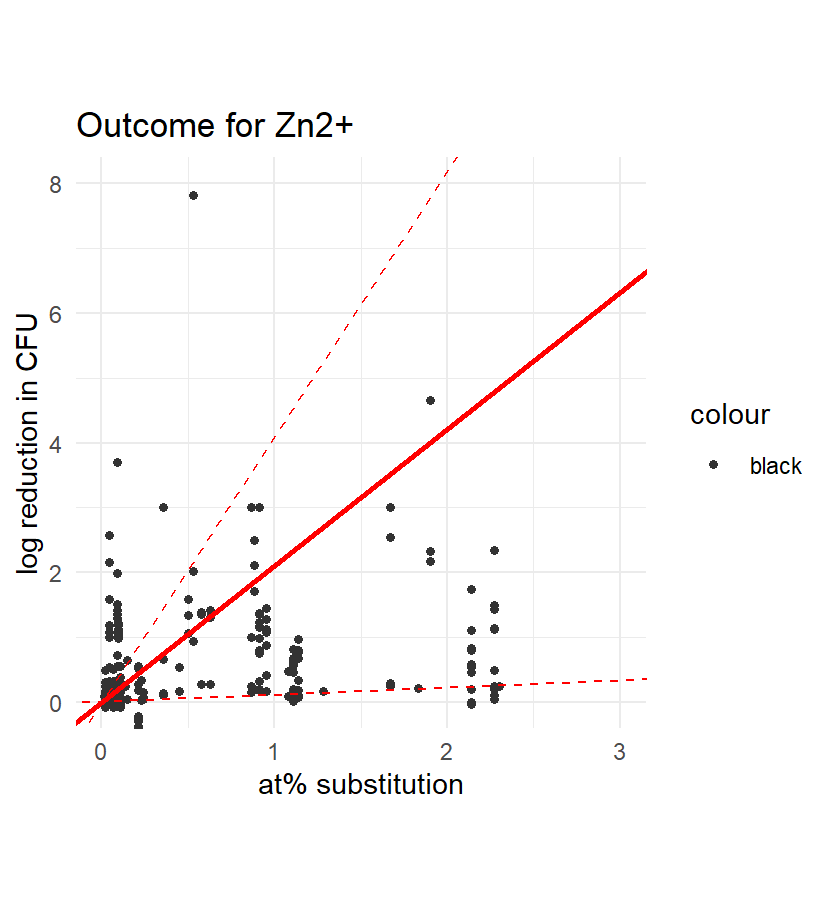

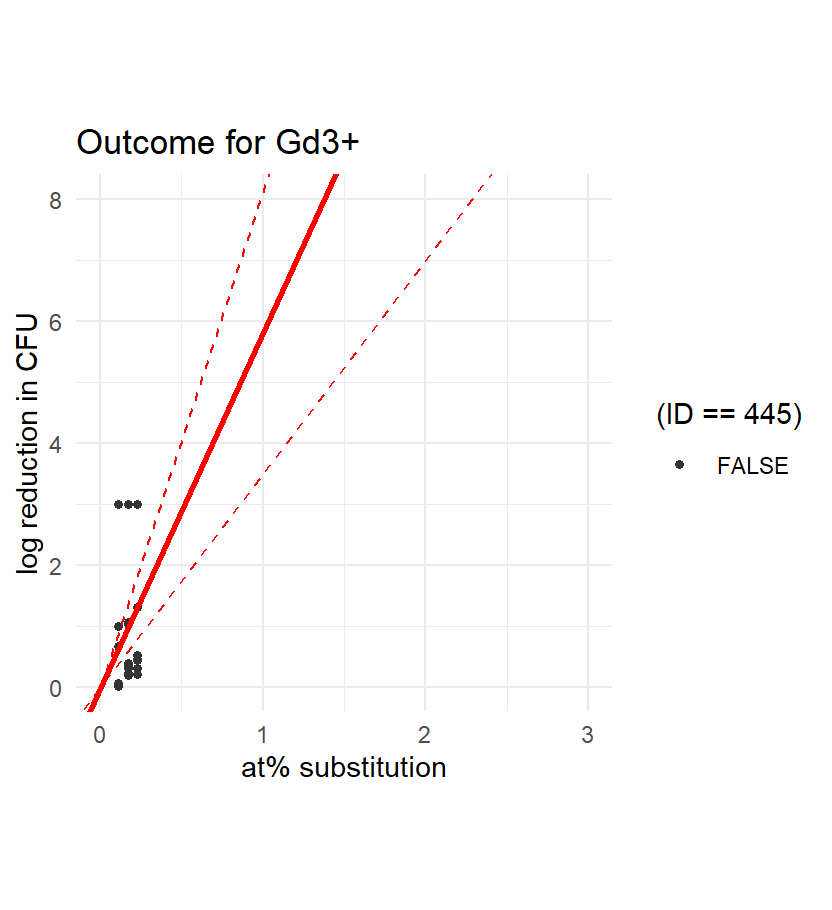

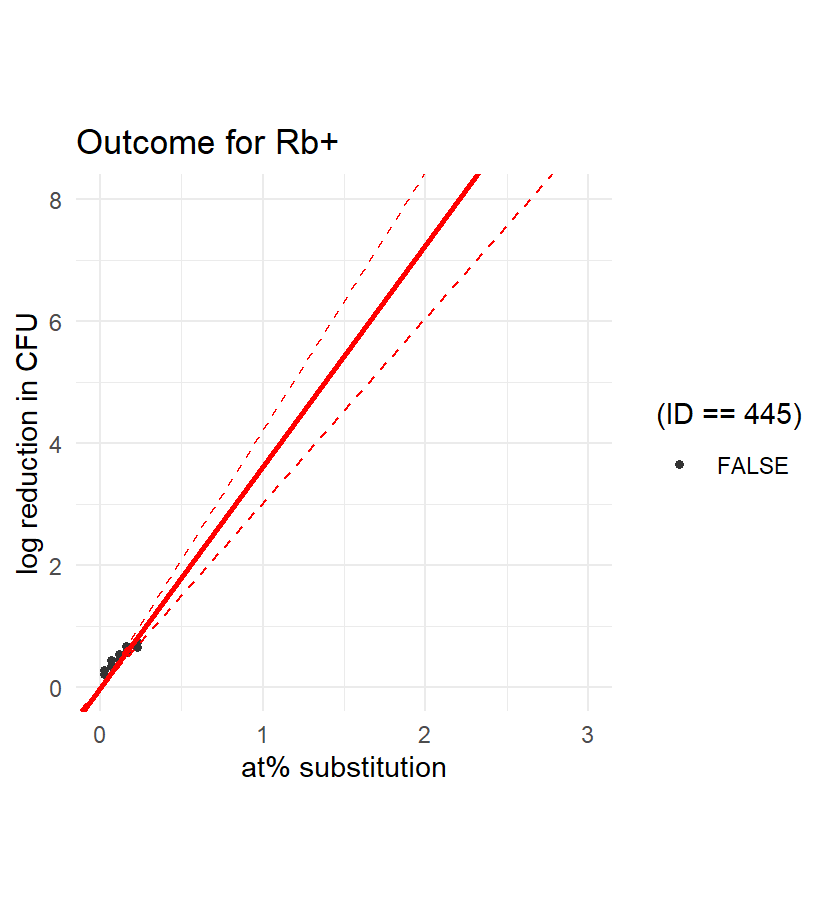

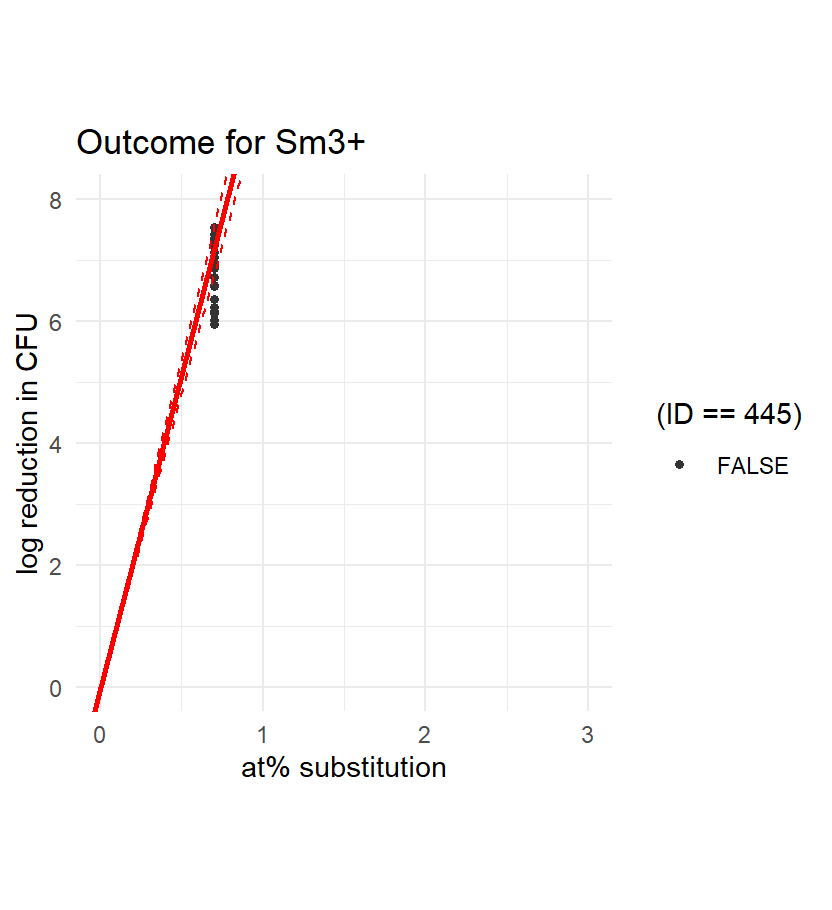


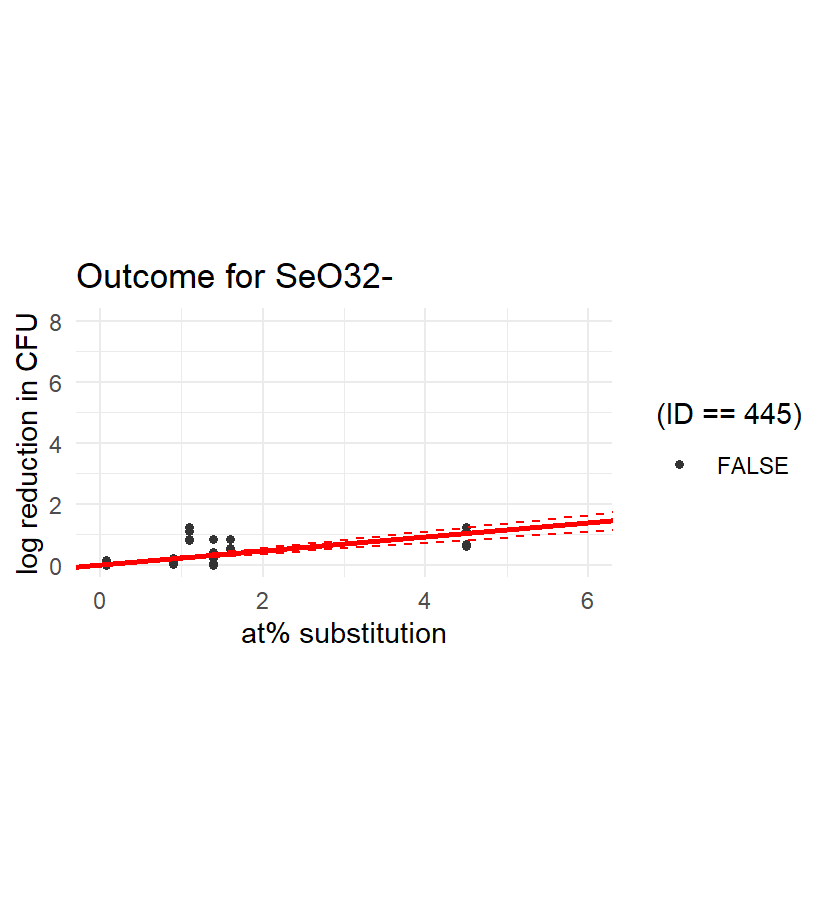


## Ions with a moderate antimicrobial effect


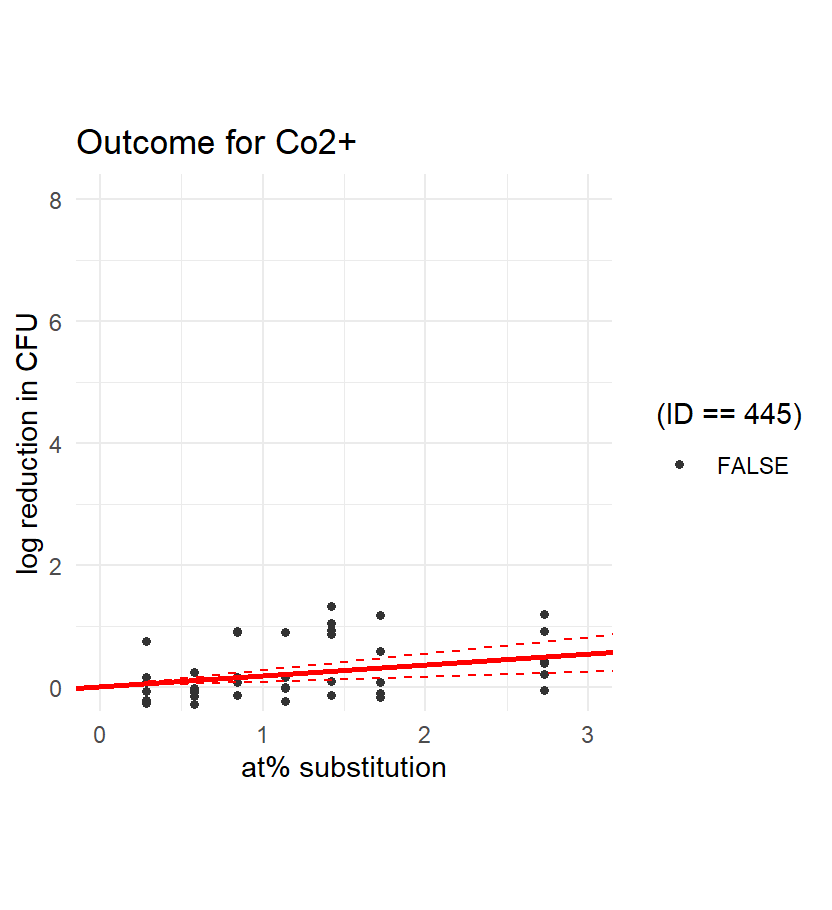

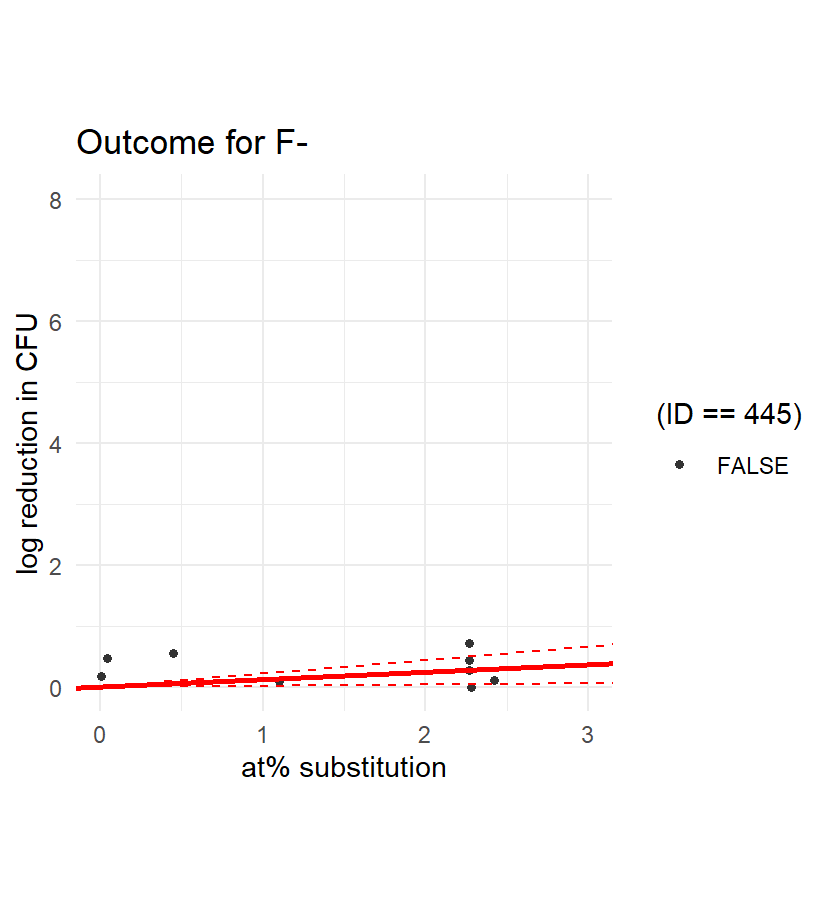

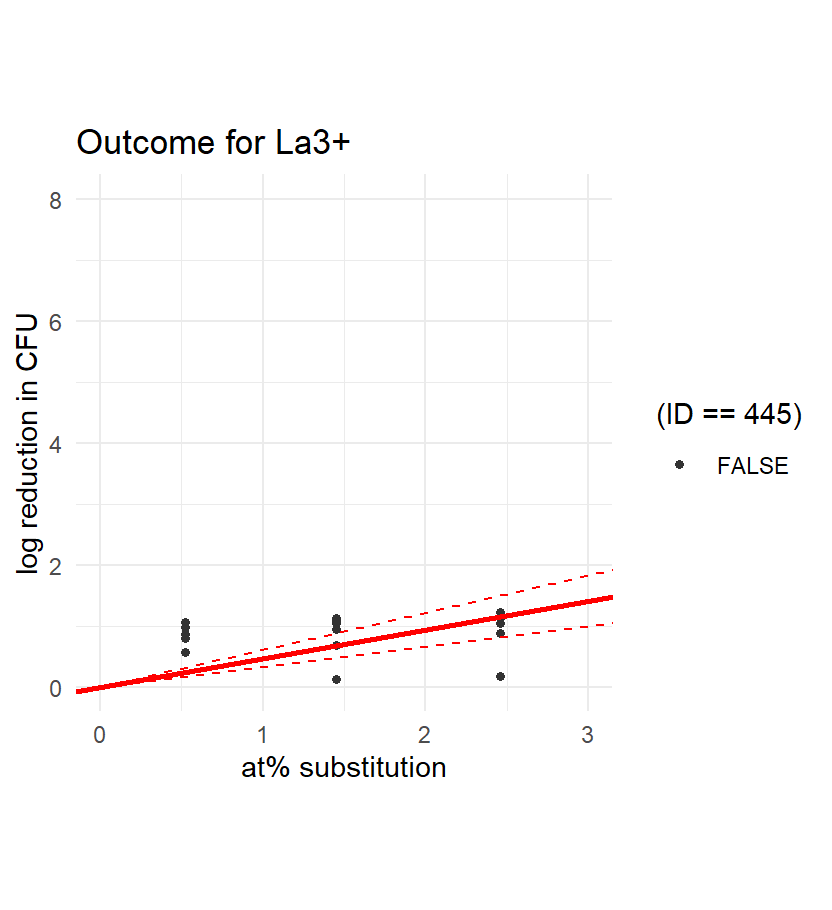

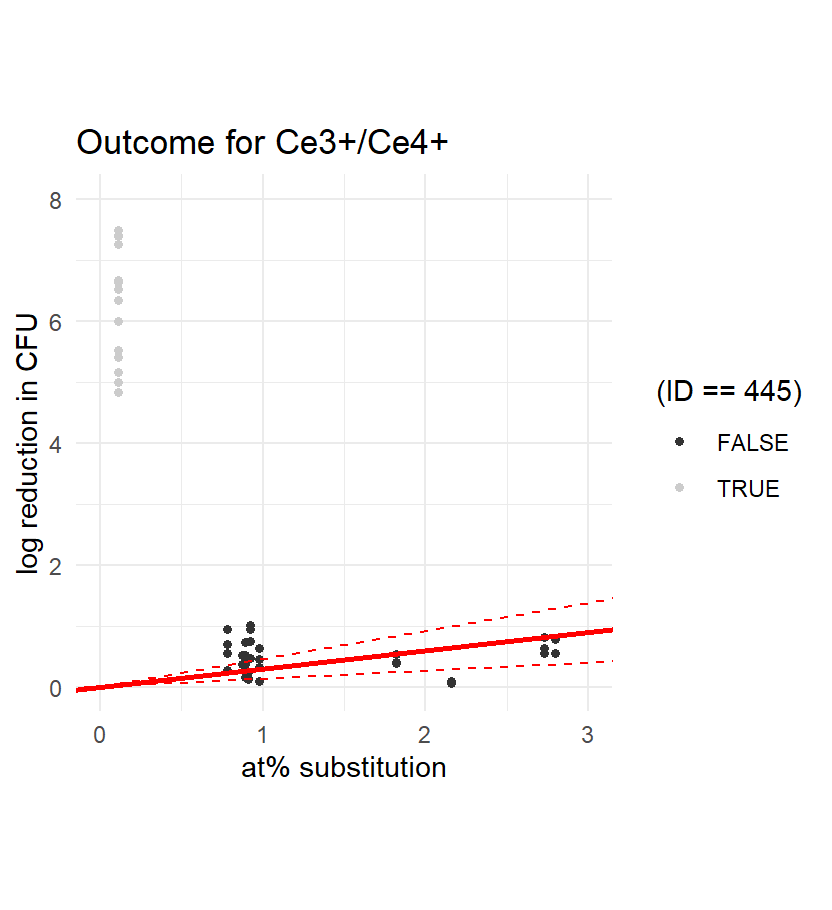

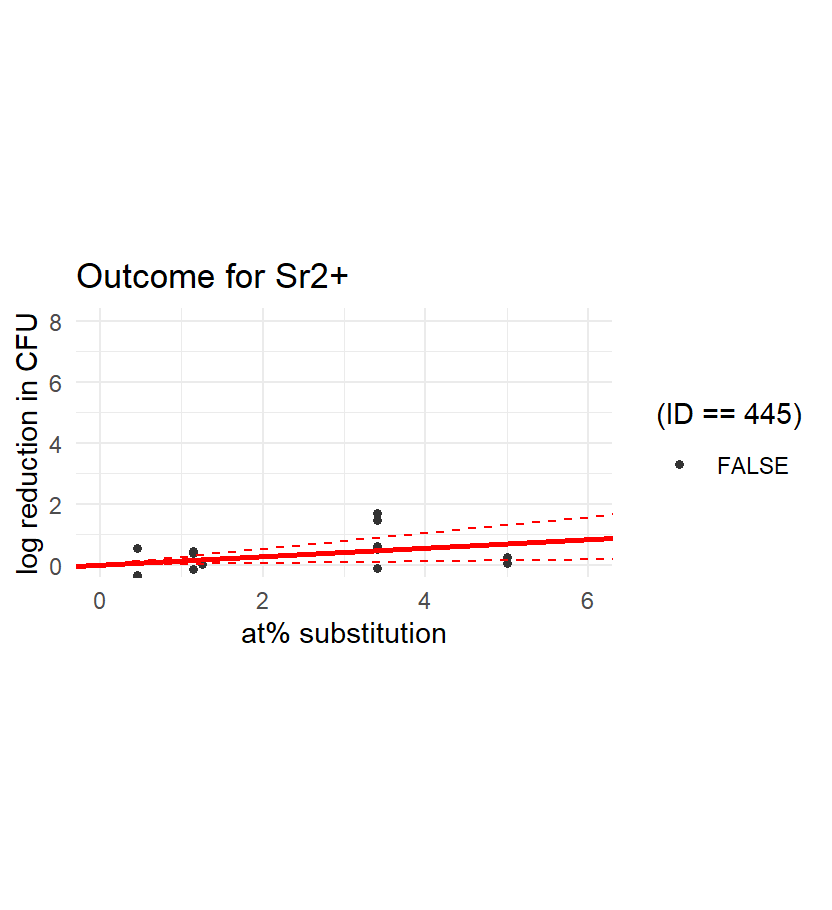


## Ions without clear antimicrobial effect

##
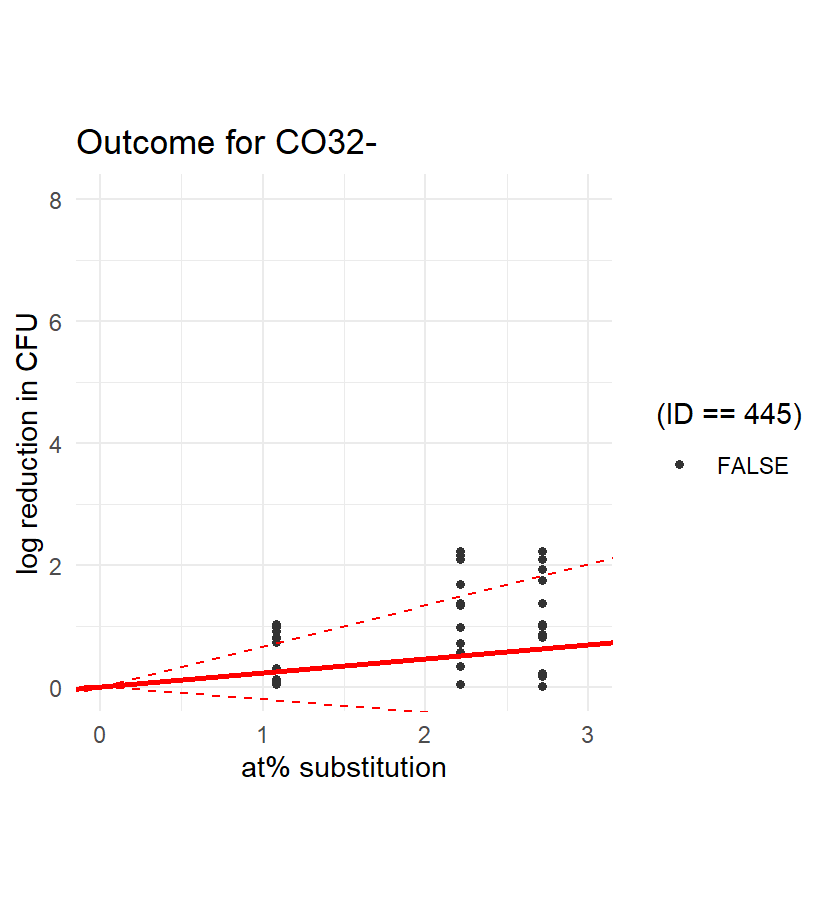

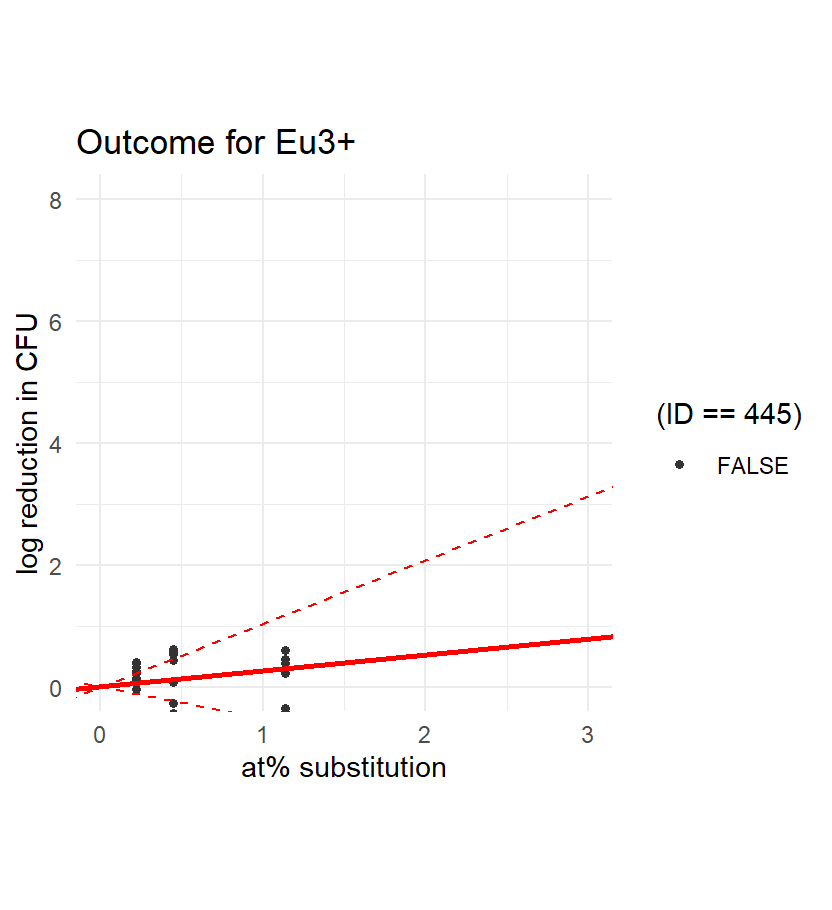

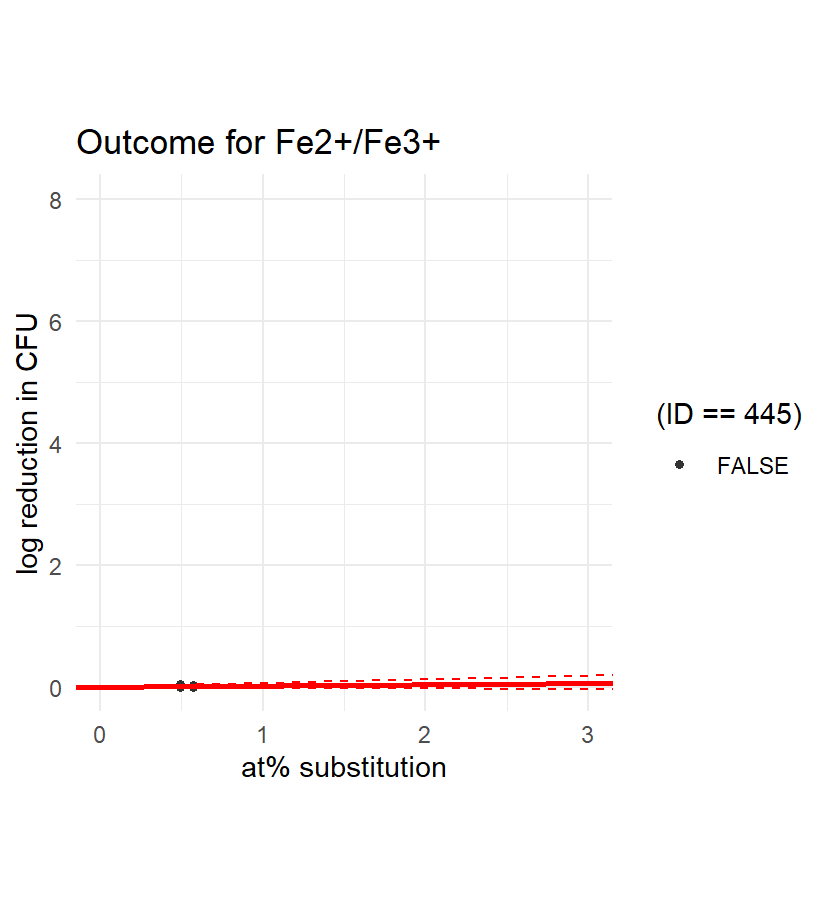

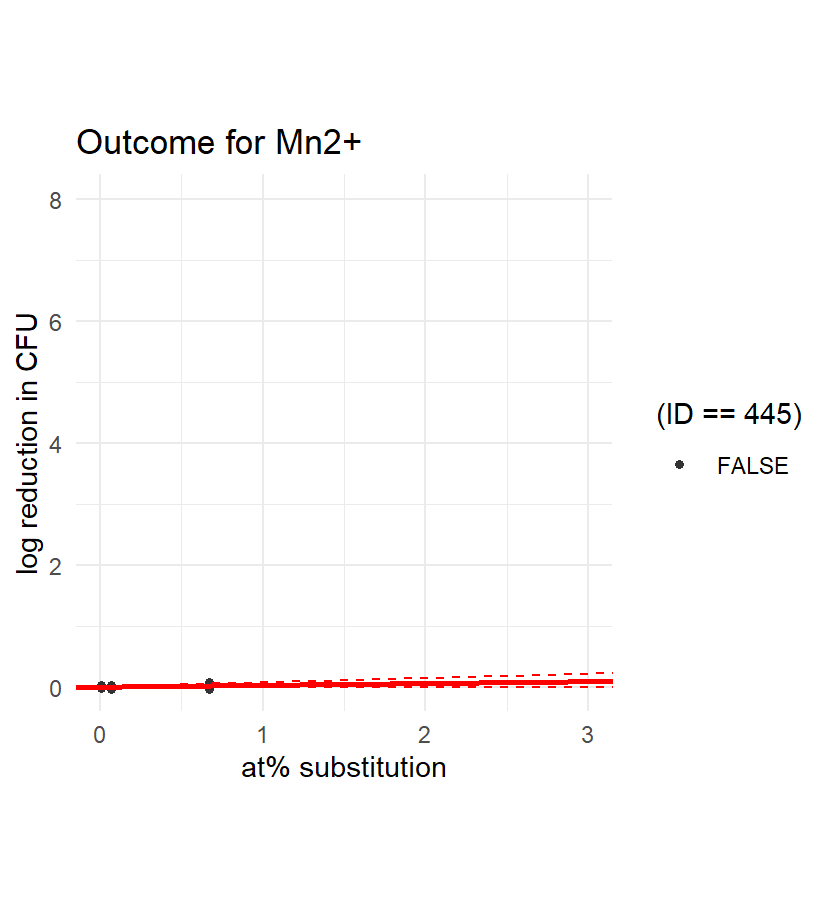

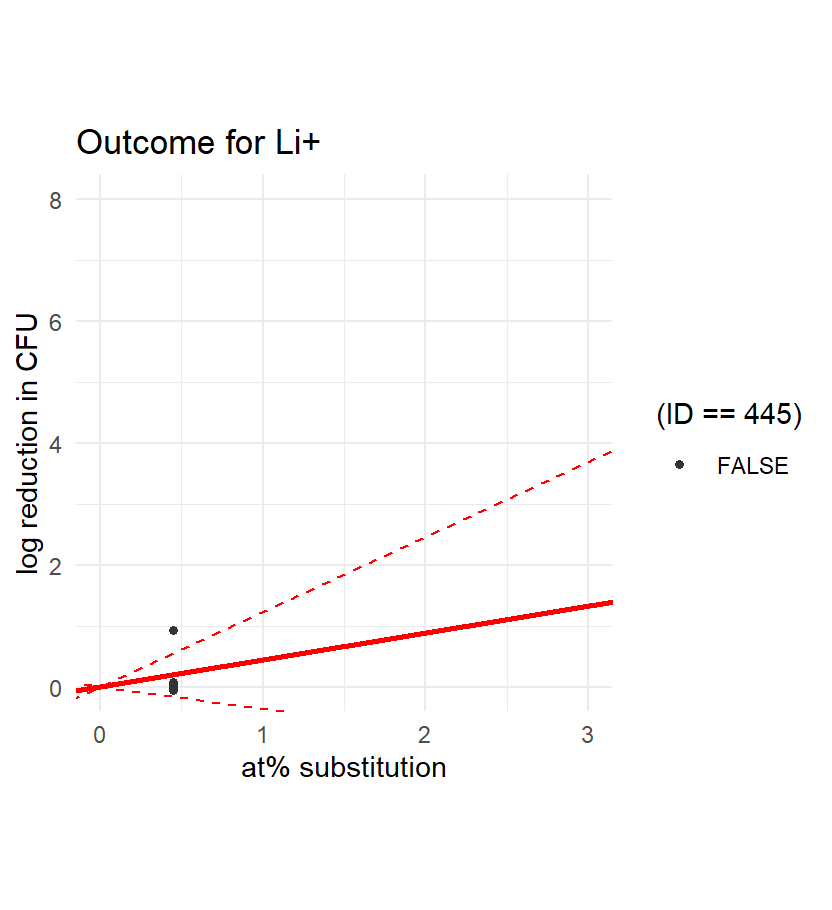

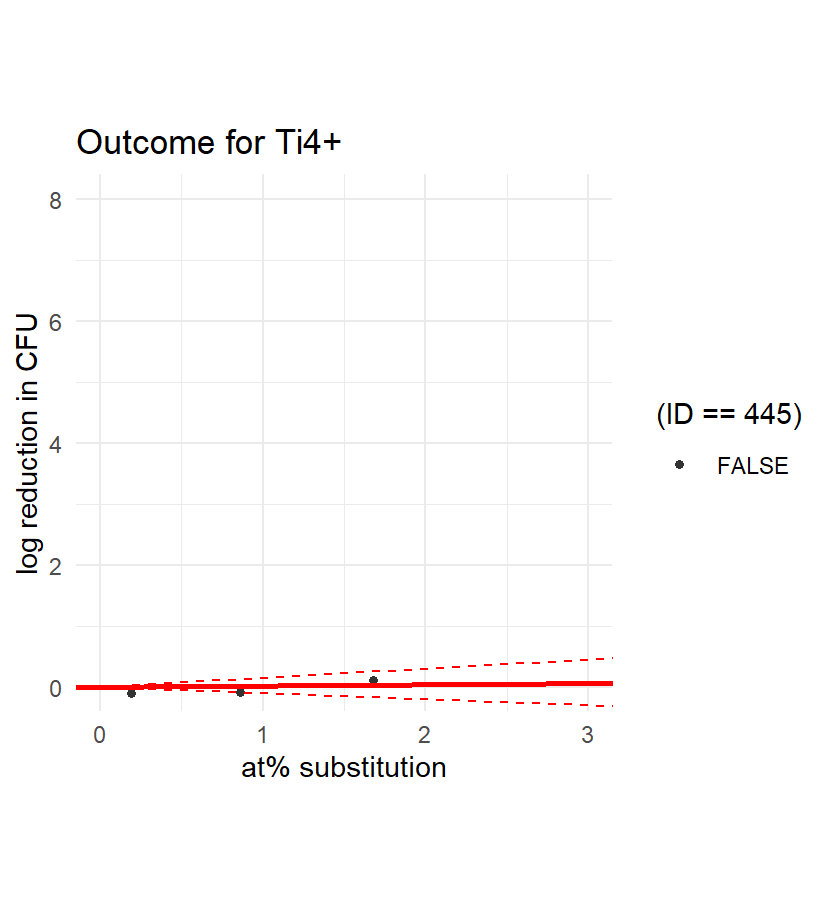


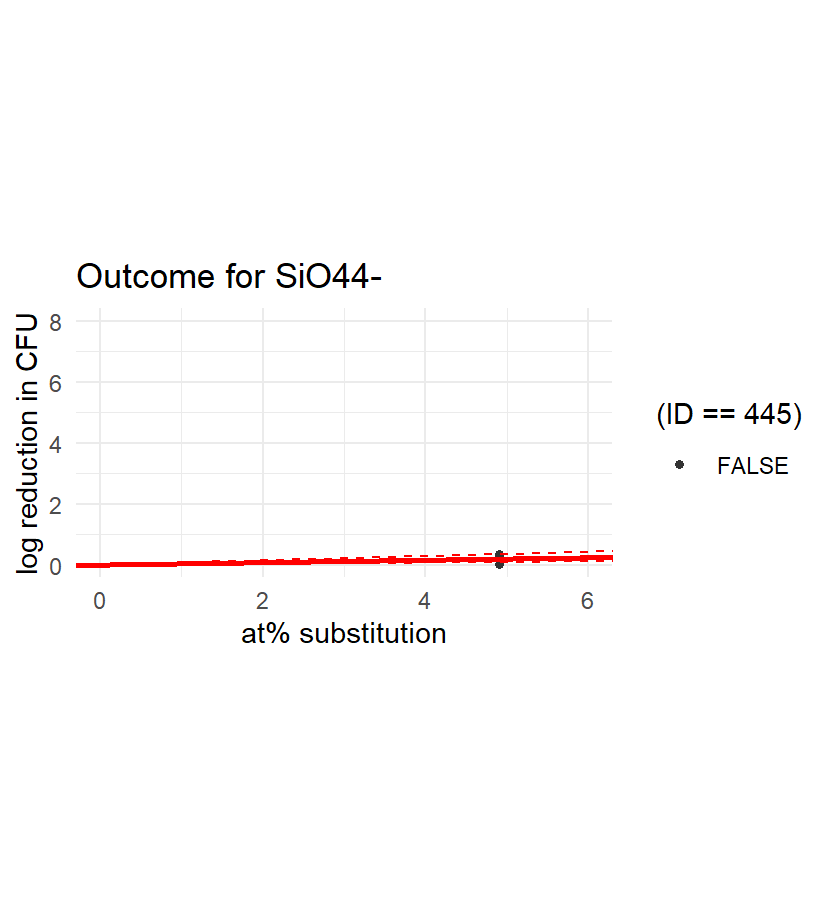

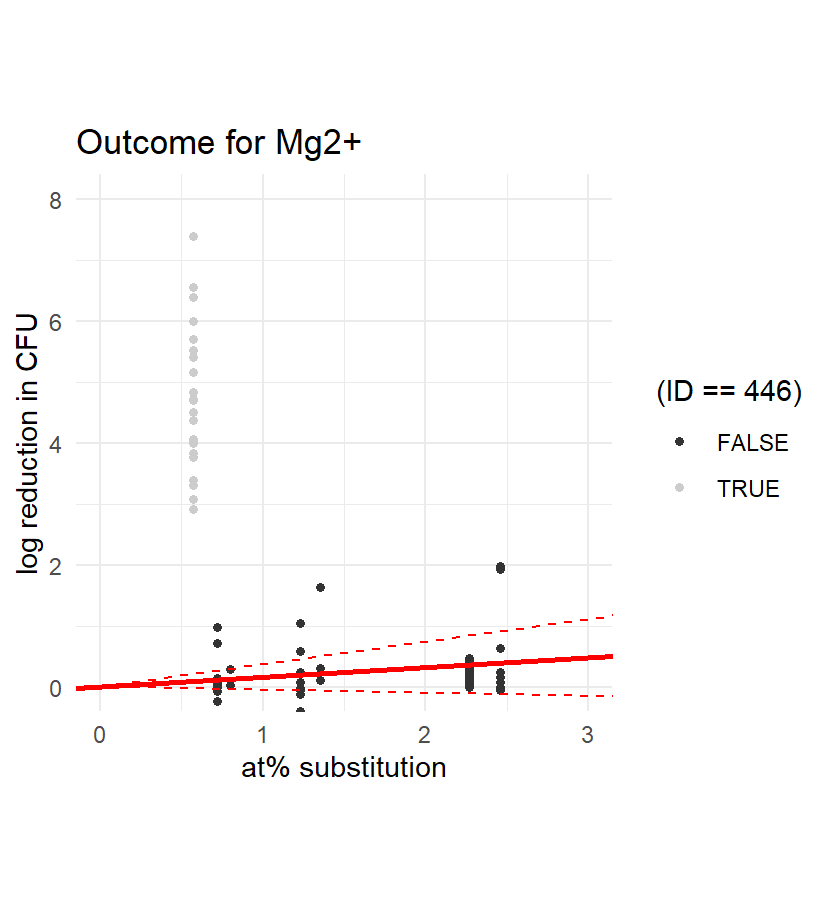

Supplement: Appendix E - RoB assessment [file mmc4.docx]
