# Supplementary material for: Antimicrobial activity of ion-substituted calcium phosphates: A systematic review
Source: Heliyon. 2023 May 26;9(6):e16568. doi: 10.1016/j.heliyon.2023.e16568 (PMC10248076; doi:10.1016/j.heliyon.2023.e16568)
Supplement: Multimedia component 6 [file mmc6.docx]

## Risk of Bias Assessment

The risk of bias of each included study was scored from 0 (high risk of bias) to 18 (low risk of bias) based on an adapted version of the OHAT Risk of Bias Assessment tool. The scores given to the studies ranged from 6 to 16, with an average of 11.4. The full list of all studies with the resultant RoB scores can be found in appendix E.

The reason no study achieved a score of 18 was the absence of a research protocol for every included study. This resulted in a score of 1 (probably high risk of bias) for question 5, *were all measured outcomes reported?*, for most studies. Studies that neglected to report the concentration of CaP powder in the culture medium during antimicrobial testing, and studies that did not perform elemental analysis on the synthesised materials (simply reporting the ratio of starting materials instead) obtained a lower score for question 3, *can we be confident in the exposure characterisation?*. Furthermore, many studies obtained low scores for question 4, *can we be confident in the outcome assessment?* due to the reporting of bias-prone outcomes such as the inhibition zone method or the MIC/MBC with arbitrary success conditions or without the support raw data such as CFU counts or OD measurements.

## Assessing the effect of bias on the reported outcome

Studies which have a poor internal validity or studies that are subject to (publication) bias can result in a skewed picture of the antimicrobial potential of ion-substituted CaP materials. The magnitude and direction of bias on the reported outcomes was explored in two ways. Firstly, the data for silver and zinc, the two ions for which the greatest amount of data is available, was divided into two groups based on their RoB score (one group including studies with a score of <=11, the other with studies scored >=12). The predicted slopes and 95% confidence intervals for the subgroups are shown in Table 1. A correlation was found between a high score, corresponding to a high degree of internal validity and a low risk of bias, and a higher measured outcome. However, there is an increase in the variance, most likely caused by the decrease in the amount of data in each subgroup.

| **Ion** | **Slope (all)** | **Slope  (< 12)** | **Slope  (=> 12)** | **95% CI (all)** | | | **95% CI (< 12)** | | | **95% CI (=> 12)** | | |
| --- | --- | --- | --- | --- | --- | --- | --- | --- | --- | --- | --- | --- |
| **Ag+** | 7.44 | 5.14 | 12.08 | 2.42 | - | 12.46 | 1.09 | - | 9.19 | 0.22 | - | 23.93 |
| **Zn2+** | 2.11 | 0.43 | 2.96 | 0.11 | - | 4.10 | -0.01 | - | 0.88 | -0.04 | - | 5.96 |

Table 1: The predicted antimicrobial slopes (in log CFU reduction per at% substitution)for silver- and zinc substituted CaP as a function of RoB score.

Secondly, the effect of publication bias was estimated by constructing funnel plots and estimating the extend of missing (unpublished results) using the trim-and-fill method (see Figure 1). Each point in the funnel plots corresponds to a single study, for which the slope and standard error was predicted using a simple linear model with intercept 0. The trim-and-fill method assesses the funnel plot for asymmetry and estimates the effect size without publication bias by adding ‘virtual studies’ to symmetrise the funnel plot. Asymmetry in the funnel plot is an indication of publication bias and was observed in the data for both Silver and zinc- substituted CaP. The direction of publication bias is towards higher antimicrobial effect. The magnitude of the bias was estimated at 15% for both zinc and silver, meaning that the slopes predicted by our models are likely 15% higher than they would be, had there been no publication bias.


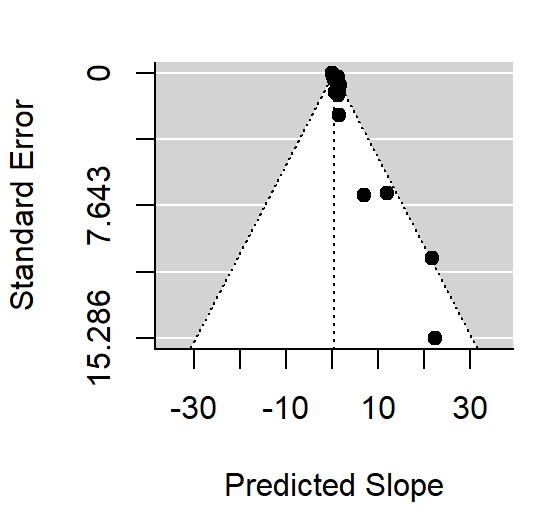

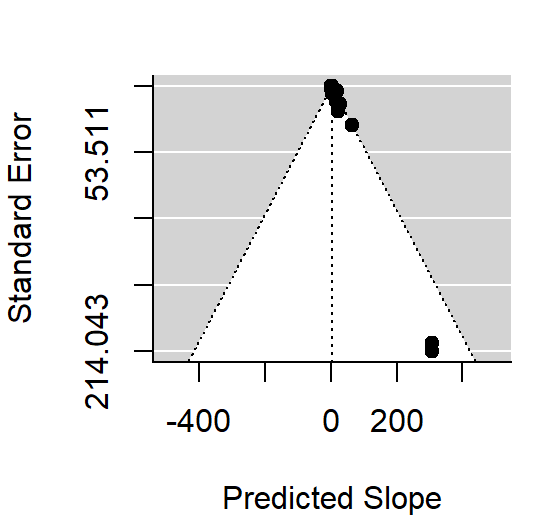


**Funnel Plot for Ag+**

**Funnel Plot for Zn2+**

Figure 1: Funnel plots constructed for silver- and zinc-substituted CaP materials.
